# Supplementary material for: MYOD-SKP2 axis boosts tumorigenesis in fusion negative rhabdomyosarcoma by preventing differentiation through p57Kip2 targeting
Source: Nat Commun. 2023 Dec 15;14:8373. doi: 10.1038/s41467-023-44130-0 (PMC10724275; doi:10.1038/s41467-023-44130-0)
Supplement: Supplementary file 1 — Supplementary Information [file 41467_2023_44130_MOESM1_ESM.pdf]

Supplementary Figure 1

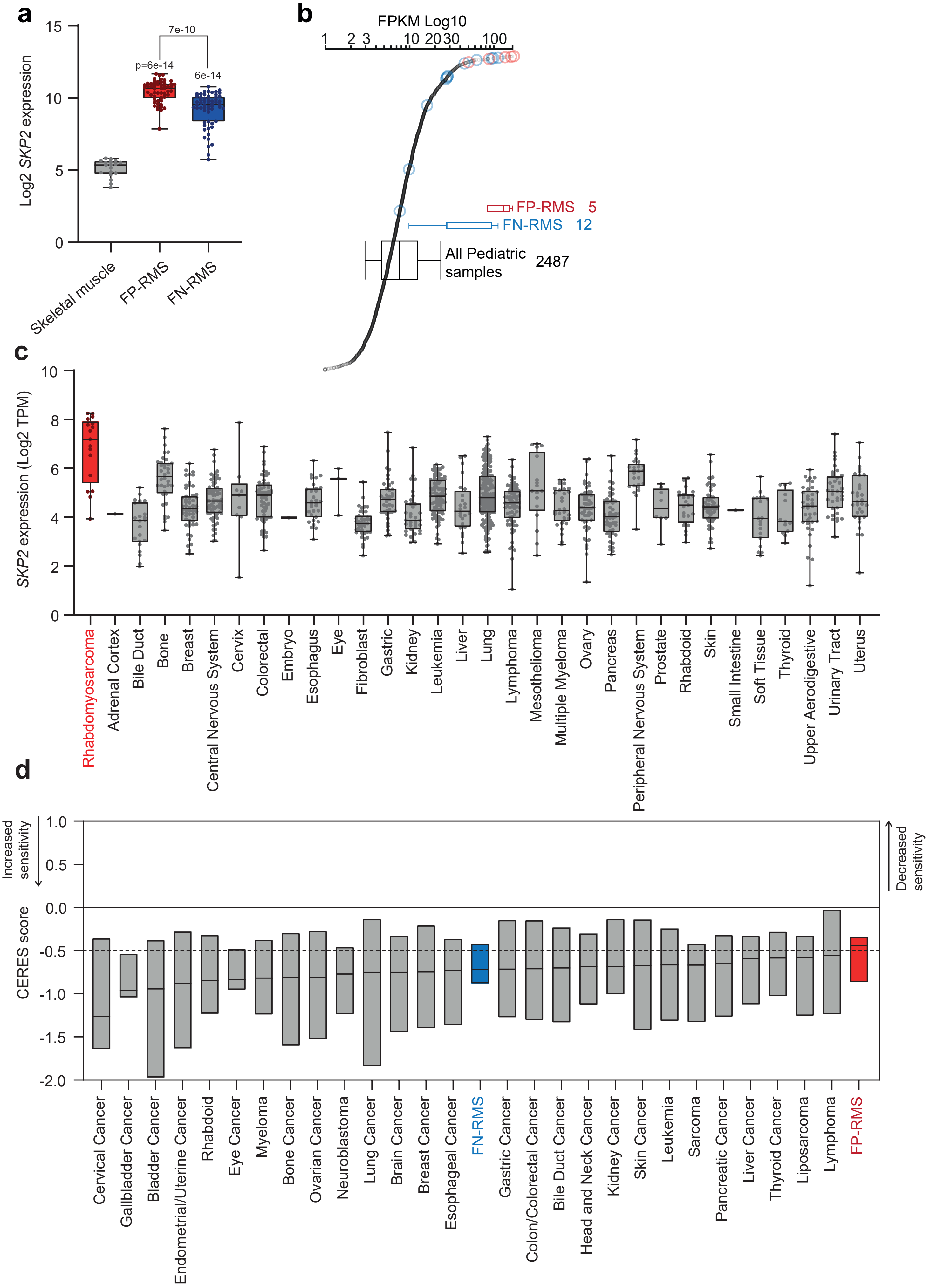

Supplementary Figure 1. SKP2 is highly expressed in RMS.

a, Box plot of *SKP2* gene expression profiling of a cohort including Fusion Positive Rhabdomyosarcoma (FP- RMS) (n=54) and Fusion Negative Rhabdomyosarcoma (FN-RMS) (n=58) patients compared to skeletal muscle tissues (n=18) (see Methods) (one-way ANOVA). Box plots show 25th to 75th quartiles, black bar shows the median, and whiskers go down to the smallest value and up to the largest **b,** Scatter plot for RNA-seq data of *SKP2* expression in RMS (St. Jude PeCan database), compared to all pediatric samples (n=5 FP-RMS, n=12 FN-RMS, all Pediatric samples=2487, data represented as box and whiskers with mean +/- maxima). FPKM, Fragment per kilobase million reads. **c,** Box plot for RNA-seq data from Achilles project depicting *SKP2* expression across RMS and other tumor cell lines. TPM, Transcripts per Million. Box plots show 25th to 75th quartiles, black bar shows the median, and whiskers go down to the smallest value and up to the largest **d,** Floating bars box plot for CRISPR *SKP2* depletion from Achilles project across different tumor types, FN-RMS and FP-RMS (https://depmap.org/portal/achilles/). Data presented as the interval between minimum and maximum CERES score value, black bar shows the median. Full line identifies a score of 0 (equivalent to a gene that is not essential in a given cell line) and dashed line identifies a score of -0.5 (equivalent to a gene that is dependent in a given cell line). Source data are provided as a Source Data file.

Supplementary Figure 2

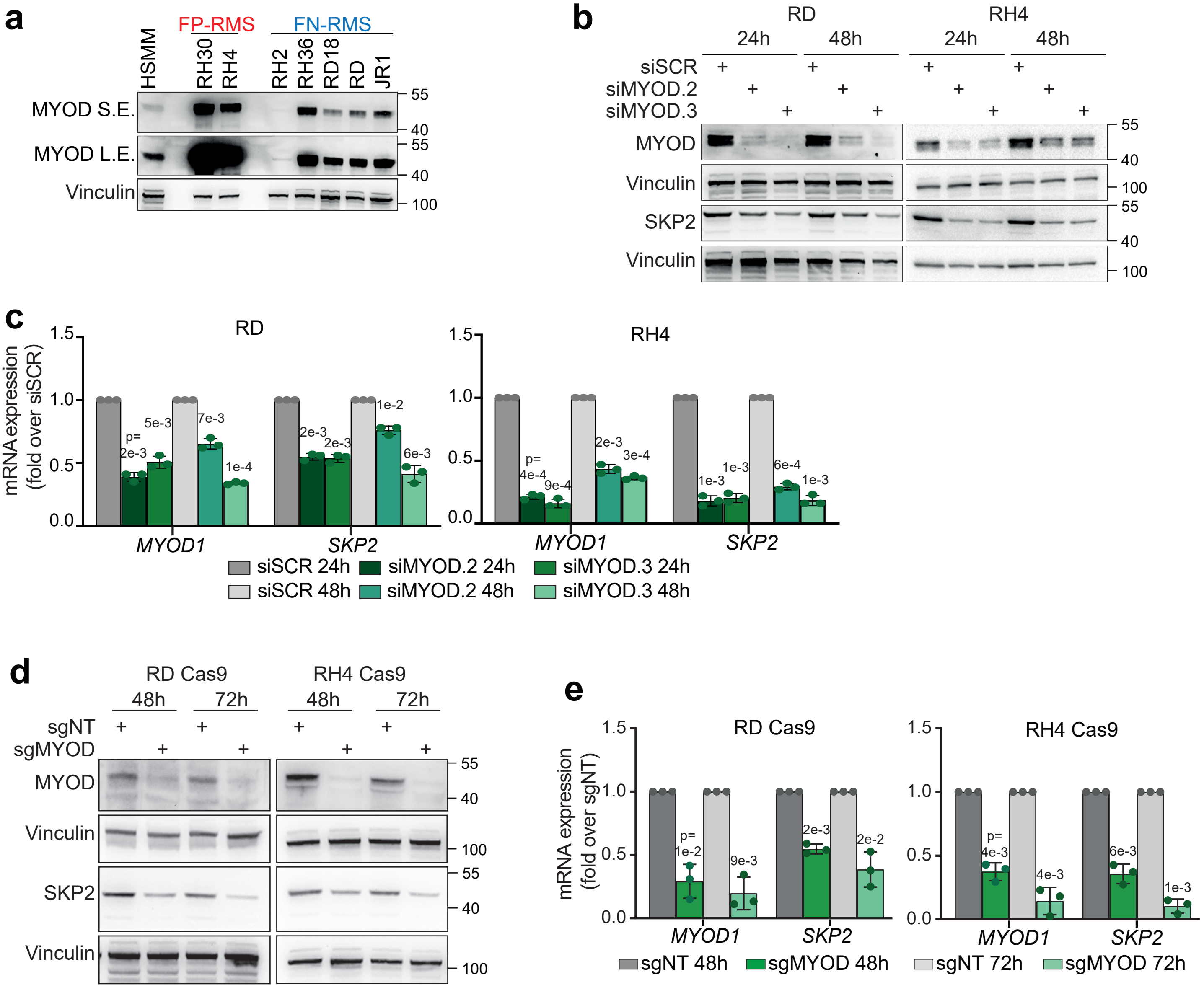

Supplementary Figure 2. SKP2 is transcriptionally regulated by MYOD.

a, Representative western blot (n = 3 independent experiments) of the indicated proteins in HSMM (normal control cells), FP-RMS and FN-RMS cell lines. Vinculin is the loading control. **b,** Representative western blot (n = 3 independent experiments) of the indicated proteins on RD and RH4 cells transfected with either Scrambled (siSCR) or two different MYOD siRNA sequences (siMYOD.2 and siMYOD.3) at 24 hours (h) and 48h post-transfection. Vinculin is the loading control. **c,** mRNA levels (RT-qPCR) of *MYOD1* and *SKP2* on cells treated as in (**b**) were normalized to *GAPDH* levels and expressed as fold increase over siSCR. n = 3 independent experiments, data presented as mean values ± SD, two-way ANOVA. **d,** Representative western blot (n = 3 independent experiments) of the indicated proteins on RD and RH4 Cas9 cells infected with either non-targeting (sgNT) or MYOD (sgMYOD) sgRNA-expressing vector at the reported time points. Vinculin is the loading control. **e**, mRNA levels (RT-qPCR) of *MYOD1* and *SKP2* on cells treated as in (**d**) were normalized to *GAPDH* levels and reported as fold increase over sgNT. n = 3 independent experiments, data presented as mean values ± SD, Student's two-tailed t-test. Source data are provided as a Source Data file.

Supplementary Figure 3

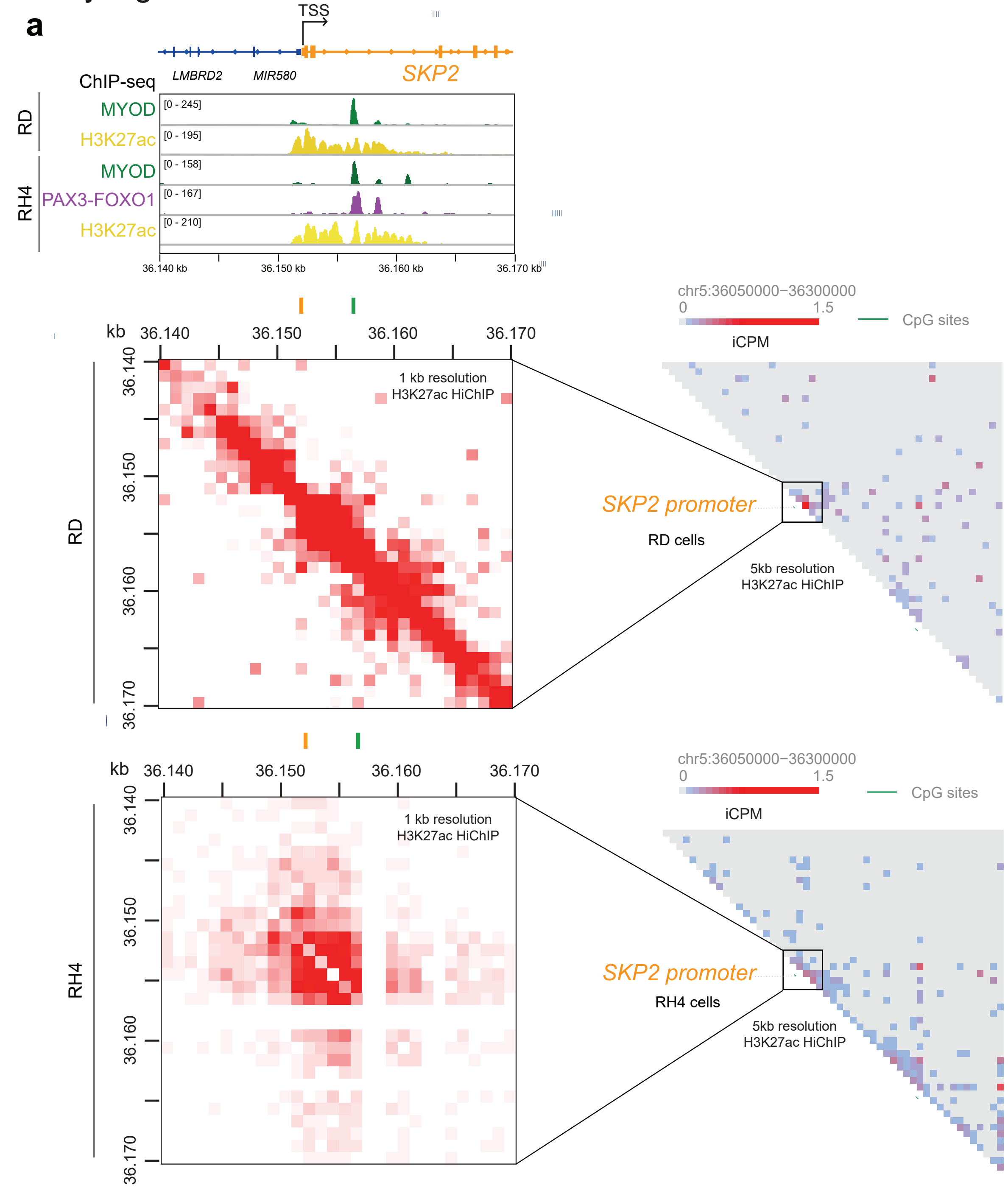

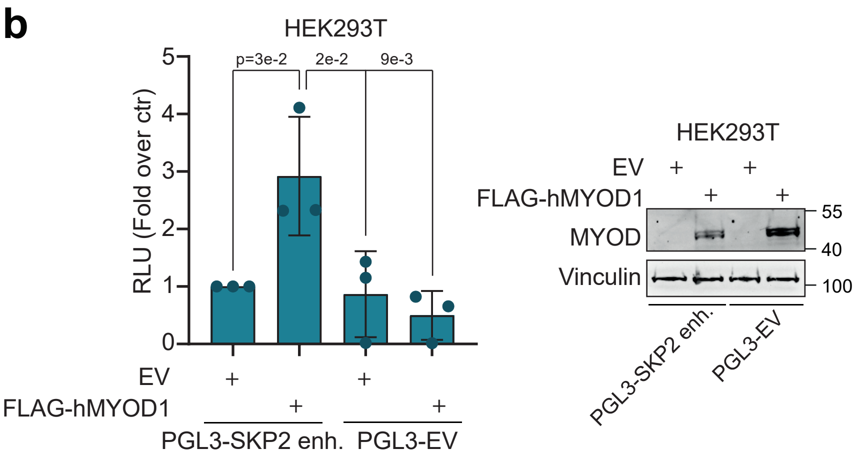

Supplementary Figure 3. MYOD bound enhancer regulates *SKP2* by directly interacting with its promoter.

a, (top) Representative profile of ChIP-seq read densities of MYOD (green), H3K27ac (yellow) and PAX3-FOXO1 (red) at *SKP2* locus on RD and RH4 cells. (bottom) RD and RH4 HiChIP analyses show chromatin interactions between *SKP2* promoter (orange vertical bar) and MYOD-bound intronic enhancer site (green vertical bar) (left, 1kb resolution); (right) HiChIP contacts that encompass *SKP2* locus are reported (5kb resolution). CPM, contacts per million. **b,** (left) Luciferase activity assays in HEK293T cells after transfection with either PGL3-EV or PGL3-SKP2 enhancer and MYOD-overexpressing vector (FLAG-hMYOD) or empty vector (EV) (left). Data were normalized to PGL3-SKP2 enhancer + EV and expressed as fold increase. n = 3 independent experiments, data presented as mean values ± SD, one-way ANOVA. (right) Representative western blot (n = 3 independent experiments) of the indicated protein on HEK293T cells treated as above. Vinculin is the loading control**.** Source data are provided as a Source Data file.

Supplementary Figure 4

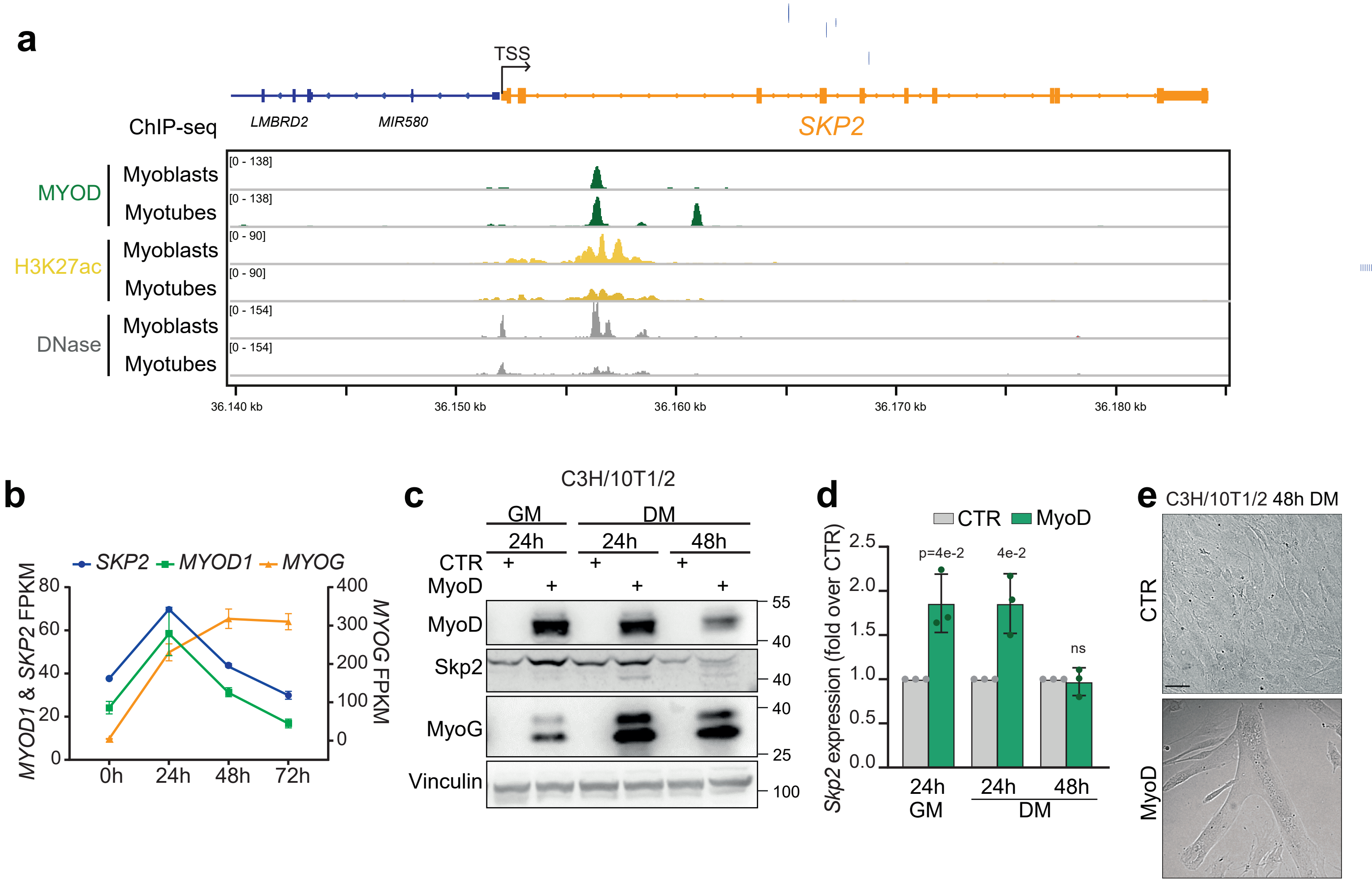

Supplementary Figure 4. SKP2 is regulated by a MYOD bound enhancer.

a, Representative profile of ChIP- seq read densities of MYOD (green) and H3K27ac (yellow) and DNase-seq (grey) data at *SKP2* locus on human myoblasts and myotubes. TSS, Transcription start site. **b,** RNA-seq analysis of *SKP2*, *MYOD1* and *MYOG* expression during HSMM differentiation. FPKM, Fragment Per Kilobase of transcript per Million mapped reads. n = 3 independent experiments, data presented as mean values ± SD **c,** Representative western blot (n = 3 independent experiments) of the indicated proteins on murine fibroblast C3H10T1/2 infected with either pBABE-puro control vector (CTR) or pBABE MyoD (MyoD) and cultured in growth medium (GM) and differentiation medium (DM) at the reported time points. Vinculin is the loading control. **d,** mRNA levels (RT- qPCR) of *Skp2* on C3H10T1/2 cultured as in (**c**) and normalized to *Hprt* levels and reported as fold increase over CTR. n = 3 independent experiments, data presented as mean values ± SD, Student's two-tailed t-test. **e,** Representative brightfield images of C3H10T1/2 cultured as in (**c**). Scale Bar = 100 µm. Source data are provided as a Source Data file.

Supplementary Figure 5

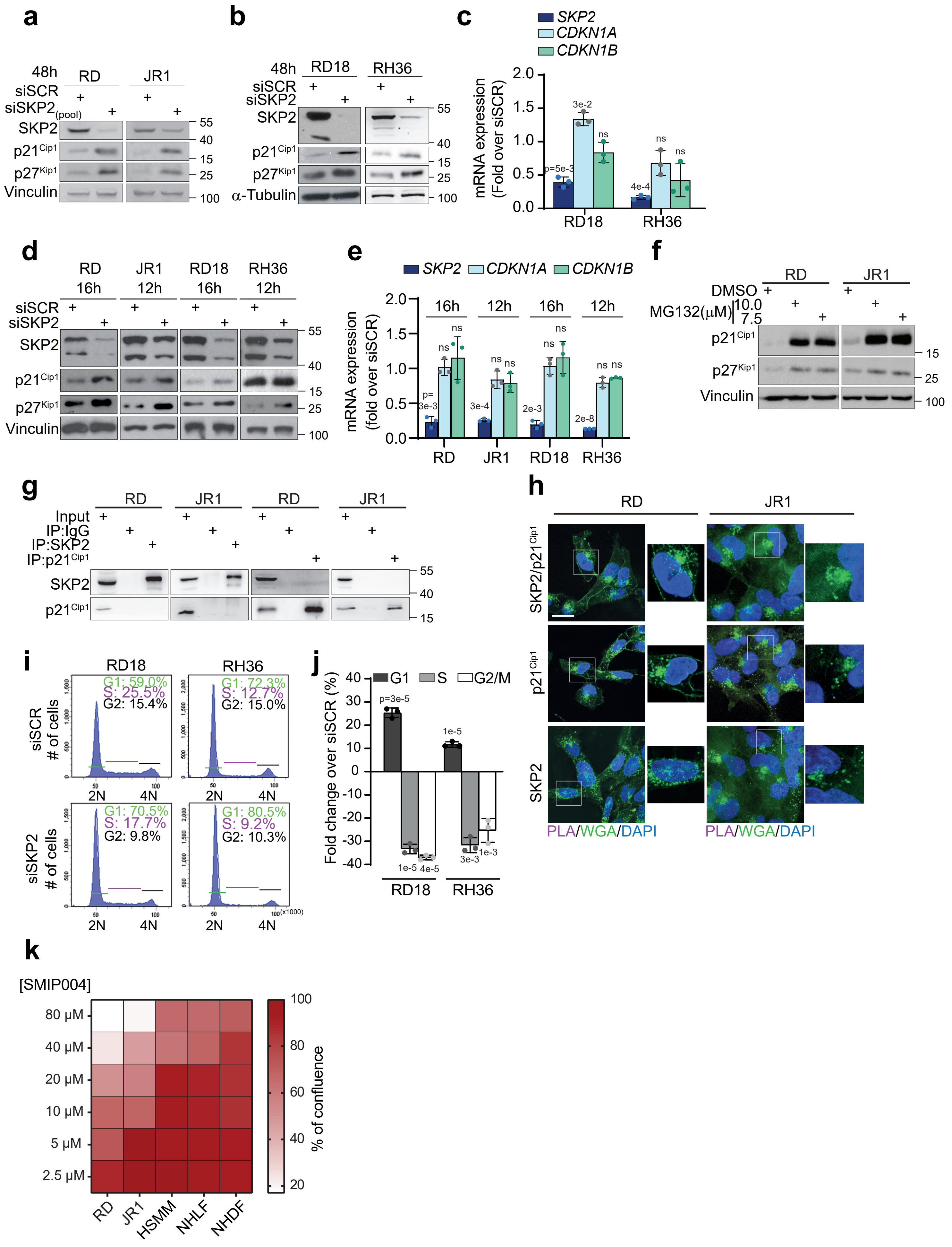

Supplementary Figure 5. SKP2 down-regulation induces cell cycle arrest in FN-RMS.

a, Representative western blot on cells transfected with Scrambled (siSCR) or SKP2 (siSKP2) siRNA smart pool. Vinculin is the loading control. **b,** Representative western blot on cells transfected with SCR or SKP2 siRNA. α-Tubulin is the loading control. **c,** RT-qPCR of *SKP2*, *CDKN1A* and *CDKN1B* in RD18 and RH36 cells treated as in (**b**) and reported as fold increase over siSCR (1 arbitrary unit, not reported). Mean values ± SD, Student's two-tailed t-test. **d,** Representative western blot on cells transfected with SCR or SKP2 siRNA at the reported time points. Vinculin is the loading control. **e,** RT-qPCR of *SKP2*, *CDKN1A* and *CDKN1B* in RD, JR1, RD18 and RH36 cells treated as in (**d**) and reported as fold increase over siSCR (1 arbitrary unit, not reported). Mean values ± SD, Student's two-tailed t-test. **f,** Representative western blot on cells treated for 8 hours with vehicle (DMSO),10 or 7.5 μM of MG132. Vinculin is the loading control. **g,** Representative western blot of co-Immunoprecipitation of either endogenous SKP2 (left) or $p21^{Cip1}$ (right) in RD and JR1 cells showing SKP2 and $p21^{Cip1}$. **h,** Representative Proximal Ligation Assay (PLA) on RD and JR1 cells. Red dots are absent indicating no SKP2/ $p21^{Cip1}$ interactions were detected. Nuclei were stained with DAPI (blue) and membranes with WGA (green). Negative controls were SKP2 or $p21^{Cip1}$ antibodies alone. Scale Bar = 100 μm. **i,** Representative diagrams of flow cytometry analysis of Propidium Iodide-stained RD18 and RH36 cells transfected with SCR or SKP2 siRNA at 48h. **j,** Histogram depicts fold changes of the percentage of siSKP2 transfected cells in G1, S and G2 phases over siSCR cells. n = 3 independent experiments, data presented as mean values ± SD, Student's two-tailed t-test. **k,** Heatmap depicting dose response effect of SMIP004 treatment for 72h on FN-RMS cells (RD and JR1), Normal Human Skeletal Muscle Myoblast (HSMM), Normal Human Lung Fibroblast (NHLF) and Normal Human Dermal Fibroblasts (NHDF). All the presented data derived from n=3 independent experiments. Source data are provided as a Source Data file

Supplementary Figure 6

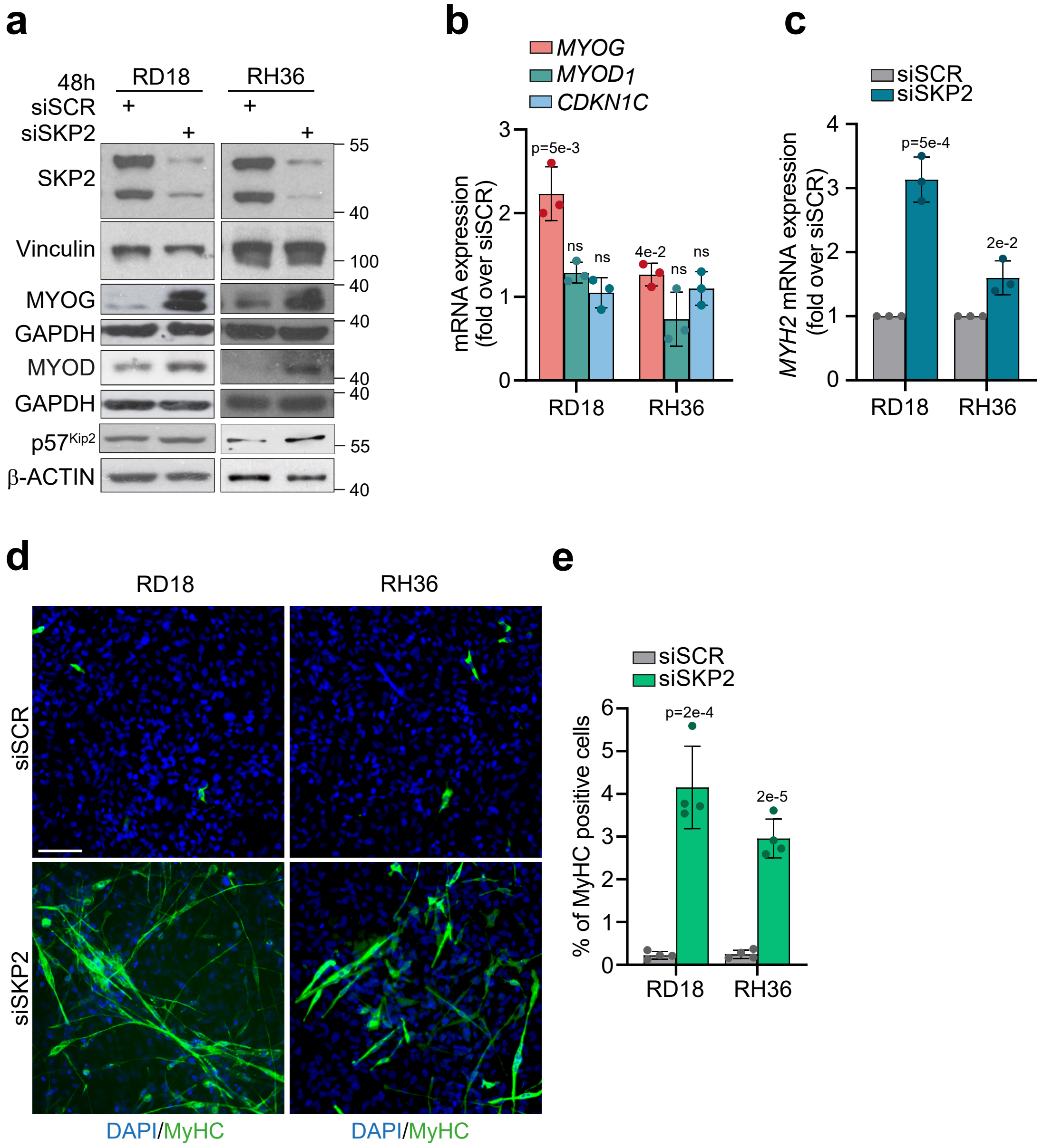

Supplementary Figure 6. SKP2 expression inhibits myogenic differentiation.

a, Representative western blot (n = 3 independent experiments) of the indicated proteins on RD18 and RH36 cells transfected with either Scrambled (siSCR) or SKP2 (siSKP2) siRNA at 48h. Vinculin, GAPDH and b-Actin are the loading controls. **b,** mRNA levels (RT-qPCR) of *MYOG*, *MYOD1* and *CDKN1C* on cells treated as in (**a**) were normalized to *GAPDH* levels and expressed as fold increase over siSCR (1 arbitrary unit, not reported). n = 3 independent experiments, data presented as mean values ± SD, Student's two-tailed t-test. **c,** mRNA levels (RT-qPCR) of *MyH2* on cells treated as in (**a**) and harvested 4 days after transfection were normalized to *GAPDH* levels and expressed as fold increase over siSCR (1 arbitrary unit). n = 3 independent experiments, data presented as mean values ± SD, Student's two-tailed t-test. **d,** Representative immunofluorescence of RD18 and RH36 cells treated as in (**a**) and cultured for 6 days, showing expression of Myosin Heavy Chain (MyHC) (green) in multinucleated fibers of siSKP2 cells. Nuclei were stained with DAPI (blue). Scale Bar = 100 µm. **e,** Histogram depicts the quantification of MyHC-positive cells treated as in (**d**). n = 4 biologically independent experiments, data presented as mean values ± SD, Student's two-tailed t- test. Source data are provided as a Source Data file.

Supplementary Figure 7

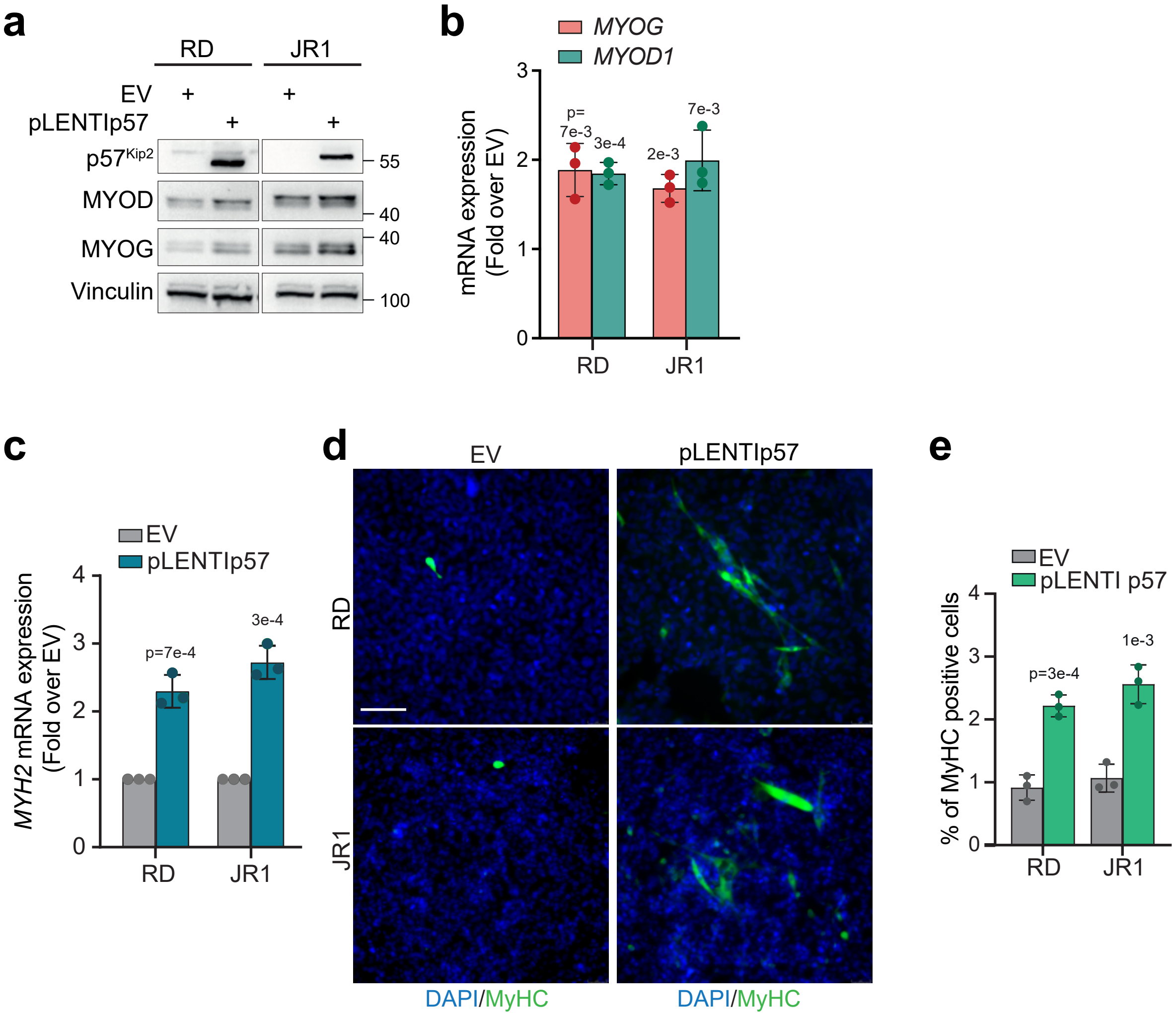

Supplementary Figure 7. SKP2 depletion-induced p57^{Kip2} increase is needed for myogenic differentiation.

a, Representative western blot (n = 3 independent experiments) of the indicated proteins on RD and JR1 cells infected with either empty vector (EV) or p57-expressing lentivector (pLENTIp57) 7 days post-selection. Vinculin is the loading controls. **b,** mRNA levels (RT-qPCR) of *MYOG* and *MYOD1* on cells treated as in (**a**) were normalized to *GAPDH* levels and expressed as fold increase over EV (1 arbitrary unit, not reported). n = 3 independent experiments, data presented as mean values ± SD, Student's two-tailed t-test. **c,** mRNA levels (RT-qPCR) of *MyH2* on cells treated as in (**a**) were normalized to *GAPDH* levels and expressed as fold increase over siSCR (1 arbitrary unit, not reported). n = 3 independent experiments, data presented as mean values ± SD, Student's two-tailed t-test. **d,** Representative immunofluorescence of RD and JR1 cells treated as in (**a**) and cultured for 6 days, showing expression of Myosin Heavy Chain (MyHC) (green) in multinucleated fibers of p57-overexpressing cells. Nuclei were stained with DAPI (blue). Scale Bar = 100 µm. **e,** Histogram depicts the quantification of MyHC-positive cells treated as in (**d**). n = 3 biologically independent experiments, data presented as mean values ± SD, Student's two-tailed t-test. Source data are provided as a Source Data file.

Supplementary Figure 8

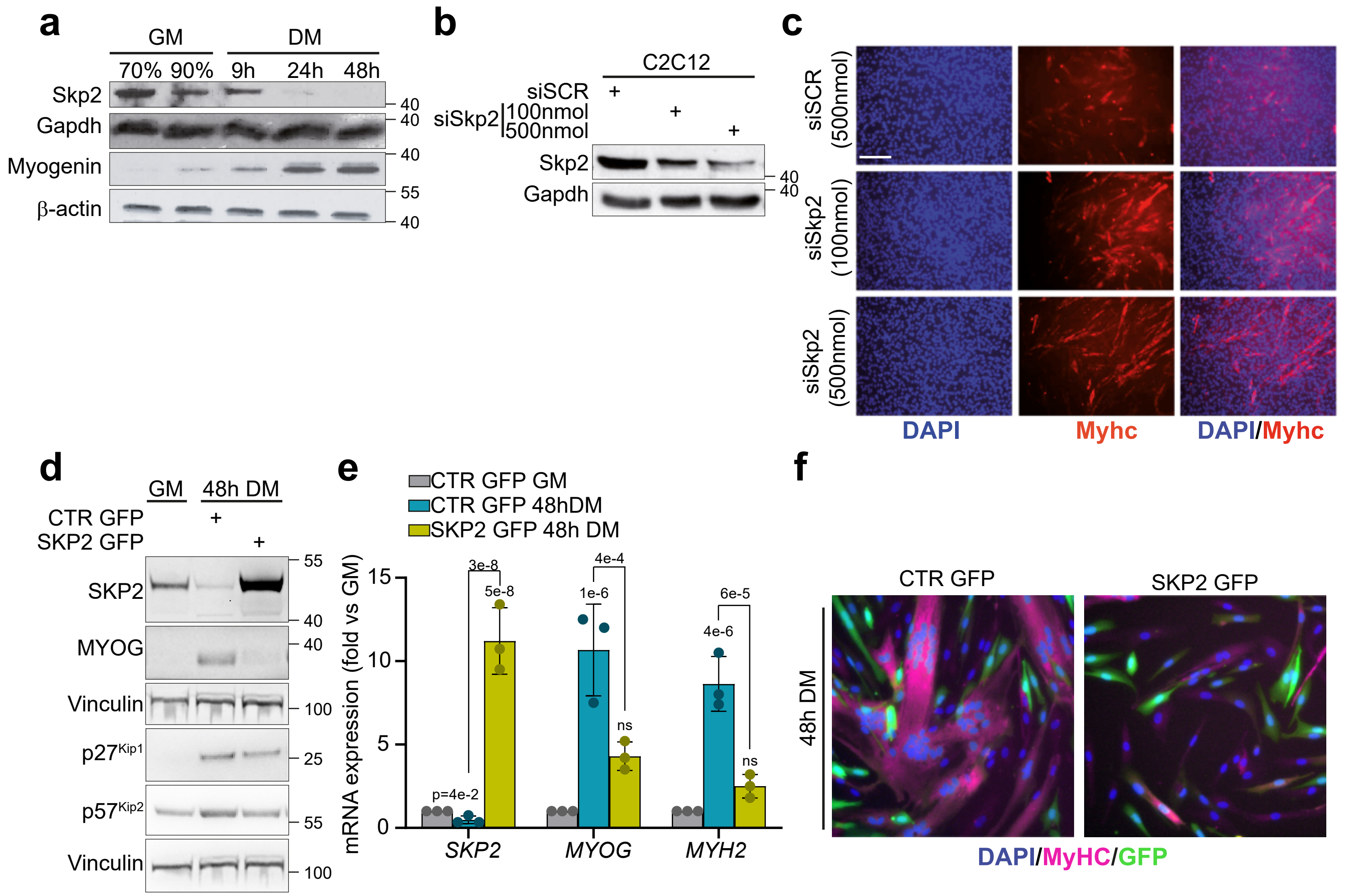

Supplementary Figure 8. SKP2 expression inhibits myogenic differentiation.

a, Representative western blot (n = 3 independent experiments) of the indicated proteins on murine myoblasts C2C12 at the indicated cell growth conditions and time points. β-actin is the loading control. GM, Growth Medium; DM, Differentiation Medium. **b,** Representative western blot (n = 3 independent experiments) of the indicated proteins on murine myoblasts C2C12 cells transfected with either Scrambled (siSCR) or Skp2 (siSkp2) siRNA at 48h. Gapdh is the loading control. **c,** Representative immunofluorescence of C2C12 cells treated as in (**b**), shifted in DM 24h post silencing and cultured for additional 2 days, showing expression of Myhc (red). Nuclei were stained with DAPI (blue). Results are representative of two independent experiments. Scale Bar = 100 µm. **d,** Representative western blot (n = 3 independent experiments) of the indicated proteins on HSMM infected with either a retrovirus co-expressing the green fluorescent protein (GFP) and the human SKP2 (SKP2 GFP) or a control GFP-expressing vector (CTR GFP) at the indicated cell growth conditions and time points. Vinculin is the loading control. HSMM, Human Skeletal Muscle Myoblasts; DM, Differentiation Medium. **e,** mRNA levels (RT-qPCR) of *SKP2, MYOG* and *MyH2* on HSMM cells treated as in (**d**) were normalized to *GAPDH* levels and expressed as fold increase over control GFP-expressing vector. n = 3 independent experiments, data presented as mean values ± SD, two-way ANOVA. **f,** Representative immunofluorescence of HSMM cells treated as in (**d**) and cultured for 2 days in DM, showing expression of MyHC (red). Nuclei were stained with DAPI (blue). Scale Bar = 100 µM. Source data are provided as a Source Data file.

Supplementary Figure 9

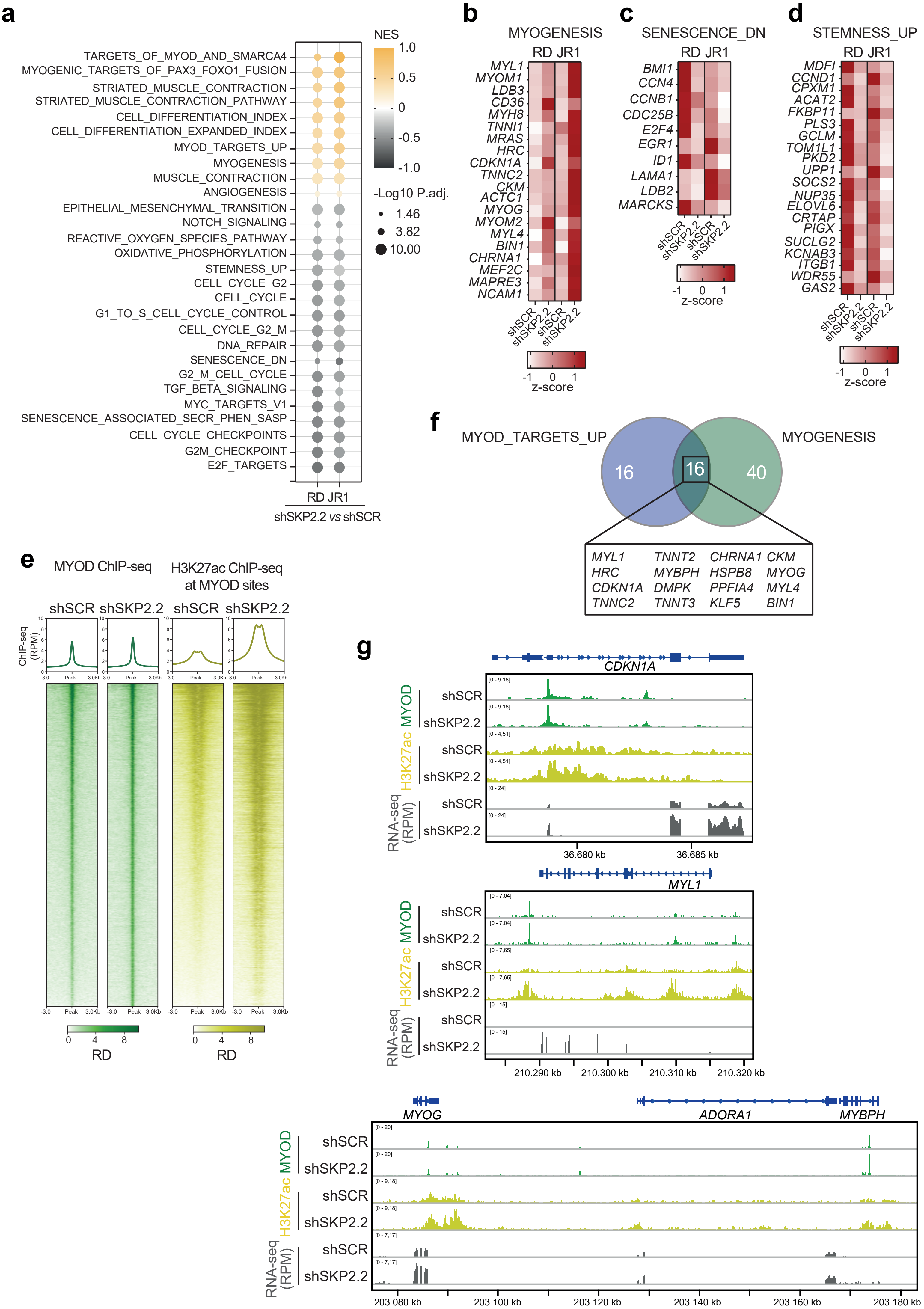

Supplementary Figure 9. SKP2 depletion impacts on transcriptional programs involved in myogenesis, senescence and stemness and induces MYOD transactivation activity.

a, Bubble plot depicting Gene Set Enrichment Analysis (GSEA) in RD and JR1 cells infected with shSKP2.2 vector. The size of the bubble is proportional to the enrichment significance (-Log10 P.adjusted), and the color of the bubble corresponds to normalized enrichment scores (NES). **b,** Heatmaps of the top 20 differentially upregulated genes belonging to MYOGENESIS gene set. Data are expressed as z-score. **c, d,** Heatmaps of top 10 and top 20 differentially downregulated genes belonging to SENESCENCE_DN and STEMNESS_UP gene sets, respectively. Data are expressed as z-score. **e,** (top) Average plots for ChIP-seq signal intensities at MYOD (left) and H3K27ac (right) peaks in RD cells. (bottom) Heatmaps of MYOD (left) and H3K27ac (right) peak intensity, with H3K27ac peaks measured at MYOD peaks. Each row represents a genomic location and is centered around MYOD peaks, extended 3Kb in each direction, and sorted by MYOD signal strength. RPM, Reads Per Million. **f,** Venn diagram depicting common upregulated genes between MYOD_TARGETS_UP and MYOGENESIS gene sets. **g,** Representative profile of ChIP-seq read densities of MYOD (green), H3K27ac (yellow) and RNA-seq (grey) at *CDKN1A, MYL1, MYOG* and *MYBPH* loci on RD cells. Source data are provided as a Source Data file.

Supplementary Figure 10

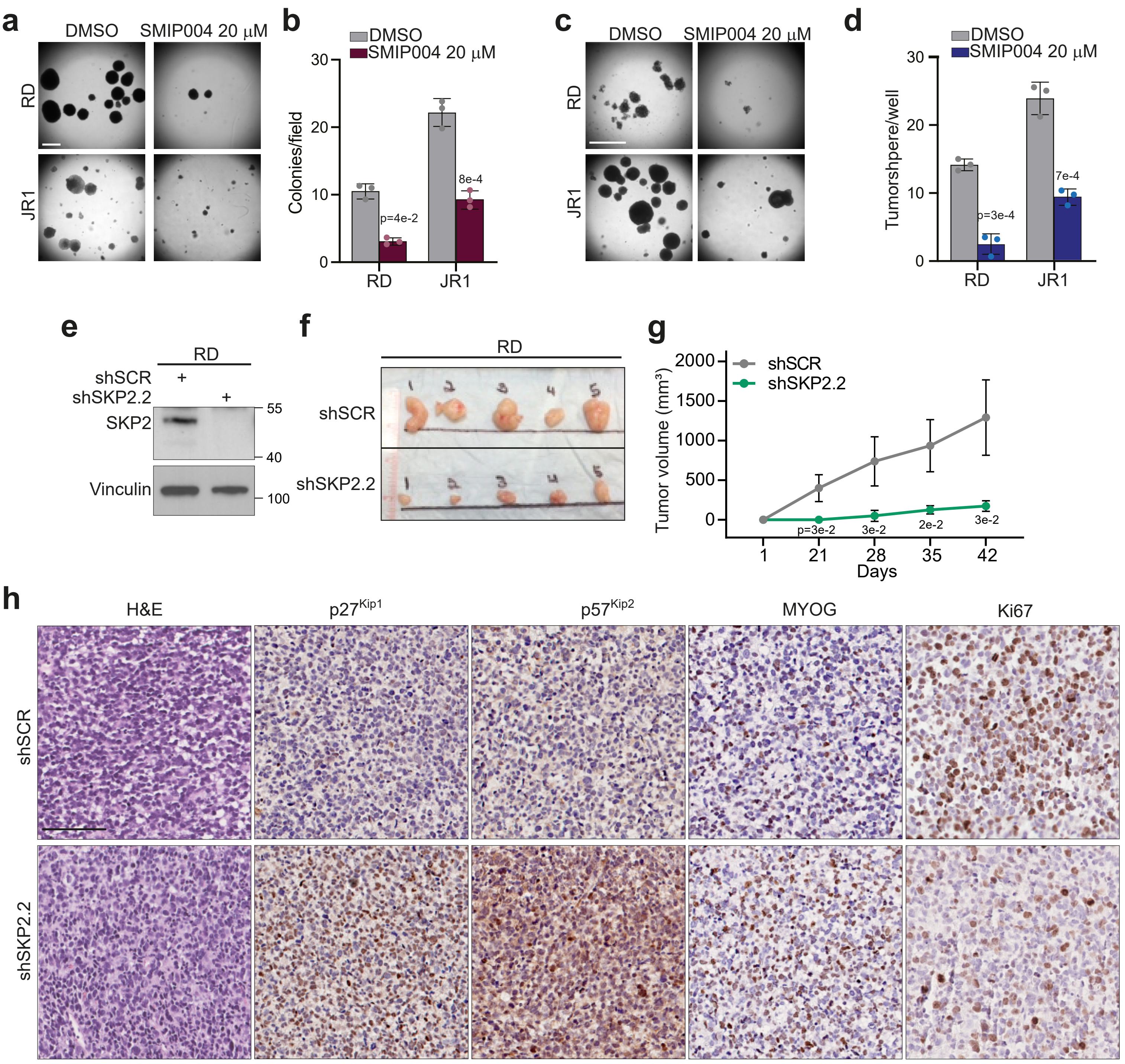

Supplementary Figure 10. SKP2 depletion reduces tumorigenicity and growth *in vitro* and *in vivo* in FN-RMS.

a, Representative light microscopy pictures of soft agar colony formation assay on RD and JR1 cells treated with SMIP004 20μM or DMSO and grown for 2 weeks. Scale bar = 50 μm. **b,** Histogram depicts the quantification of soft agar colony numbers per field. Data presented as mean values ± SD, Student's two-tailed t-test. **c,** Representative light microscopy pictures of single cell colony formation assay on RD and JR1 cells treated as in (**a**) and grown for 2 weeks. Scale bar = 50 μm. **d,** Histogram depicts the quantification of colony numbers per field. Data presented as mean values ± SD, Student's two-tailed t-test. **e,** Representative western blot of the indicated proteins on RD cells infected with either Scrambled (shSCR) or SKP2.2 (shSKP2.2) lentiviral shRNA. Vinculin is the loading control. **f,** Images of shSCR and shSKP2.2 tumors extracted from mice post euthanasia at 42 days. **g,** Tumor volume of transplanted RD expressing either shSCR (n=5) or shSKP2.2 (n=5) assessed by caliper measurement represented in mm^3 followed for 42 days. Data presented as mean values ± SD, two-way ANOVA. **h,** Representative images of H&E, $p27^{Kip1}$, $p57^{Kip2}$, MYOG and Ki67 immunohistochemistry of tumor sections from RD xenografts expressing either shSCR or shSKP2.2. Scale Bars = 100 μm. All the presented data derived from n=3 independent experiments. Source data are provided as a Source Data file.

Supplementary Figure 11

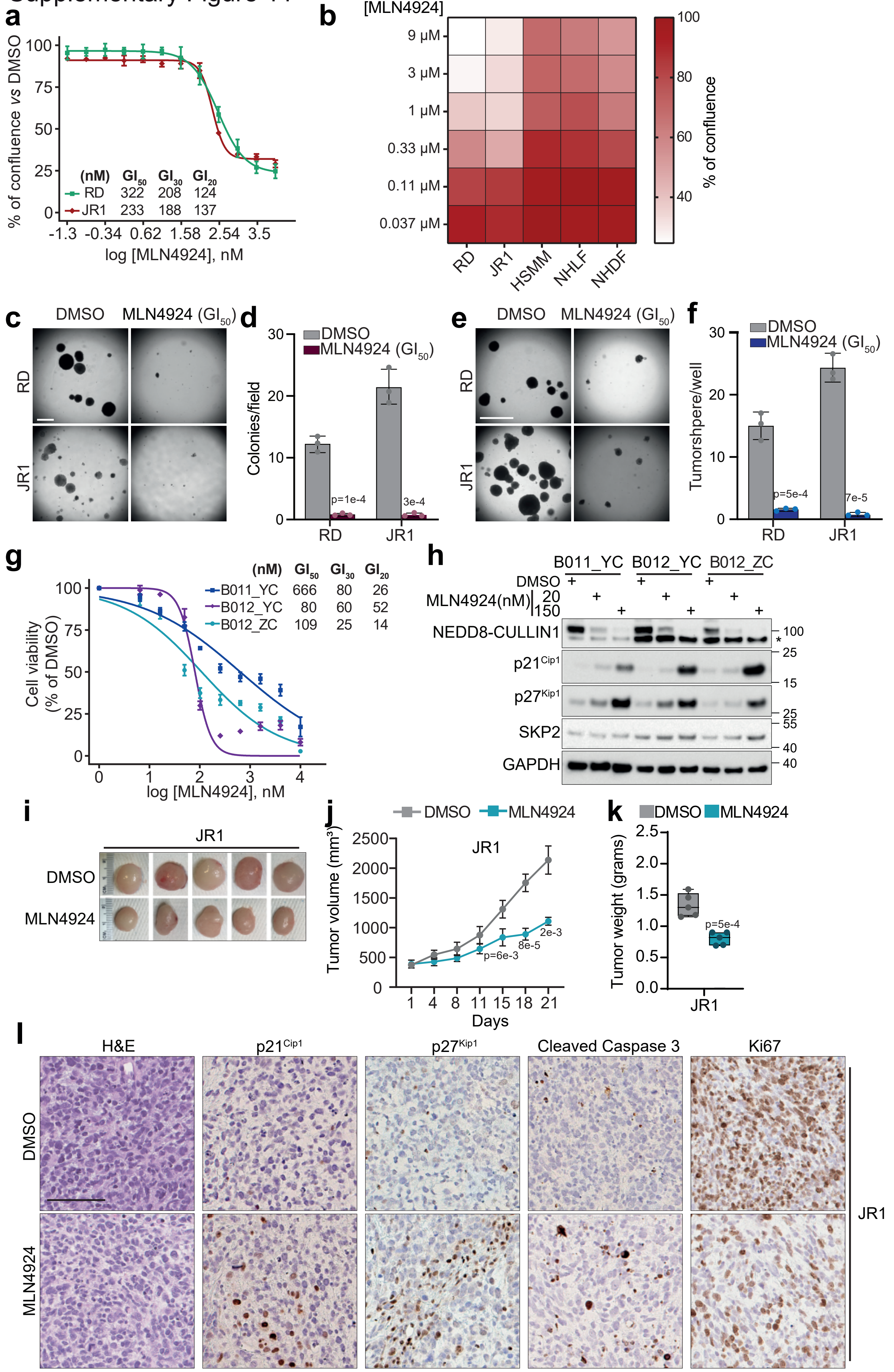

Supplementary Figure 11. MLN4924 reduces proliferation, increases caspase activity *in vitro* and impairs tumor growth *in vivo* in RMS.

a, Dose-response curves of cells treated with MLN4924. Data presented as mean values ± SEM. **b,** Heatmap depicting dose-response effect of MLN4924 treatment for 72h on FN-RMS cells (RD and JR1), Normal Human Skeletal Muscle Myoblasts (HSMM), Normal Human Lung Fibroblasts (NHLF) and Normal Human Dermal Fibroblasts (NHDF). **c,** Representative light microscopy pictures of soft agar colonies on cells treated with MLN4924 or DMSO and grown for 2 weeks. Scale bar = 50 µm. **d,** Histogram depicts the quantification of soft agar colonies. Data presented as mean values ± SD, Student's two-tailed t-test. **e,** Representative light microscopy pictures of single cell colonies of cells treated as in (**c**) and grown for 2 weeks. Scale bar = 50 µm. **f,** Histogram depicts the quantification of colonies. Data presented as mean values ± SD, Student's two-tailed t-test. **g,** Cell titer-Glo dose-response curves of 3 human PDX cell lines (B011_YC primary p53-mutated; B012_YC recurrence site A p53 wild- type; B012_ZC recurrence site B p53 wild-type) treated with MLN4924. Data presented as mean values ± SEM. **h,** Western blot on 3 human PDX cell lines treated for 72h with vehicle (DMSO) or MLN4924. GAPDH is the loading control. (*) indicates a non-specific band. **i,** Images of JR1 tumors extracted from mice treated with vehicle or MLN4924 for 3 weeks. **j,** Tumor volume of JR1 xenografts (vehicle n=5, MLN4924 n=5) treated as in (**i**) assessed by caliper measurement followed for 3 weeks treatment. Data presented as mean values ± SD, two-way ANOVA. **k,** Tumor weight of JR1 xenografts (vehicle n=5, MLN4924 n=5) treated as in (**i**). Box plots show 25th to 75th quartiles, black bar shows the median, and whiskers go down to the smallest value and up to the largest. Student's two- tailed t-test. **l,** Representative images of H&E, $p21^{Cip1}$, $p27^{Kip1}$, Cleaved Caspase and Ki67 immunohistochemistry of tumor sections from JR1 xenografts treated with vehicle or MLN4924. Scale Bars = 100 µm. All presented data derived from n=3 independent experiments. Source data are provided as a Source Data file.

Supplementary Figure 12

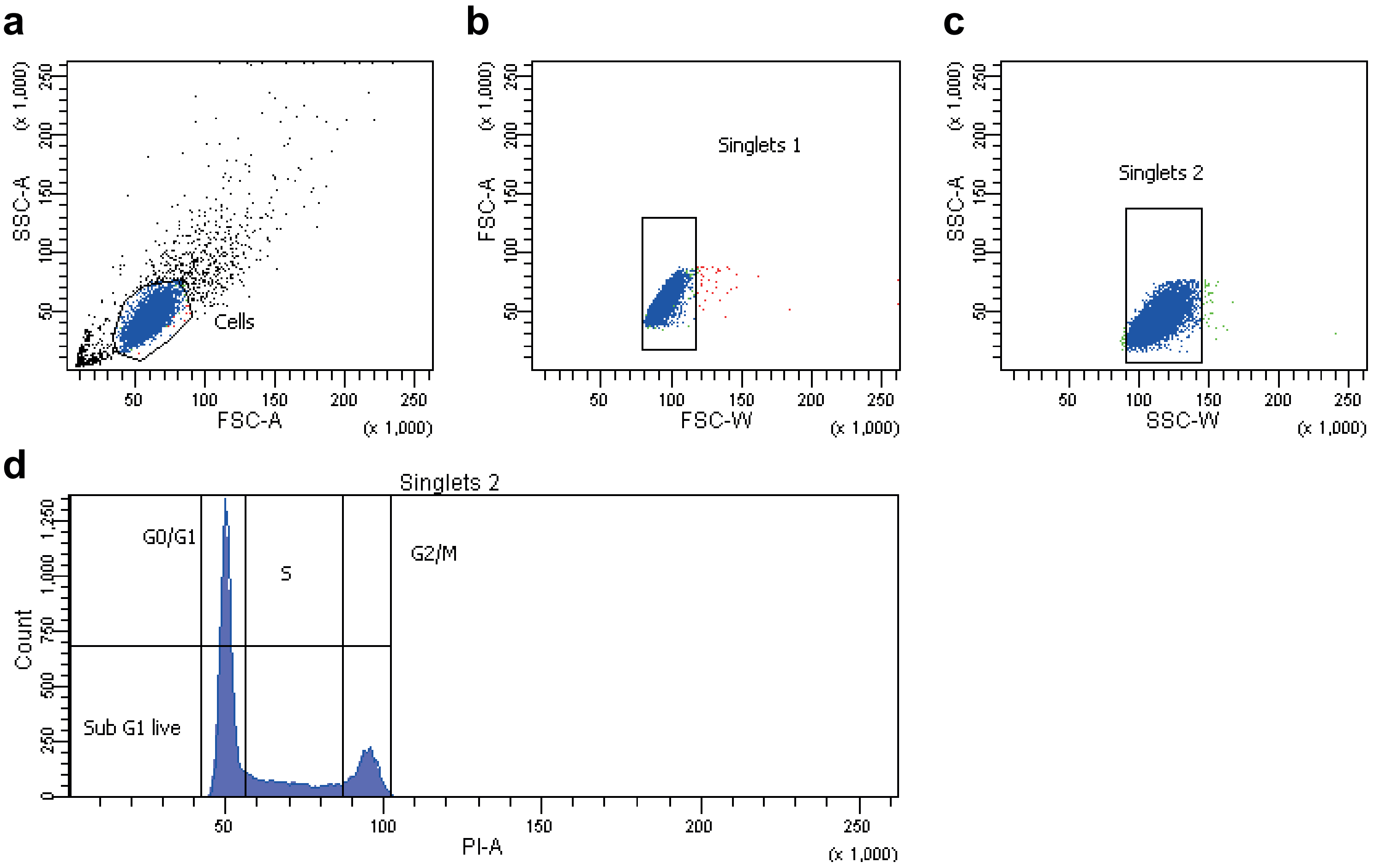

Supplementary Figure 12. Gating strategy for cell cycle analyses.

Representative gating strategy for cell cycle analyses. **a**, forward scatter and side scatter plot (FSC-A/SSC-A) for the selection of only live cells. **b**, forward scatter plot (FSC-A/FSC-W) and **c**, side scatter plot (SSC-A/SSC-W) in which all the events were gated to analyze only singlets population to avoid possible bias due to doublets. **d**, cytofluorimetric plot based on PI incorporation (DNA amount) to identify cell cycle phases.

Figure S2a

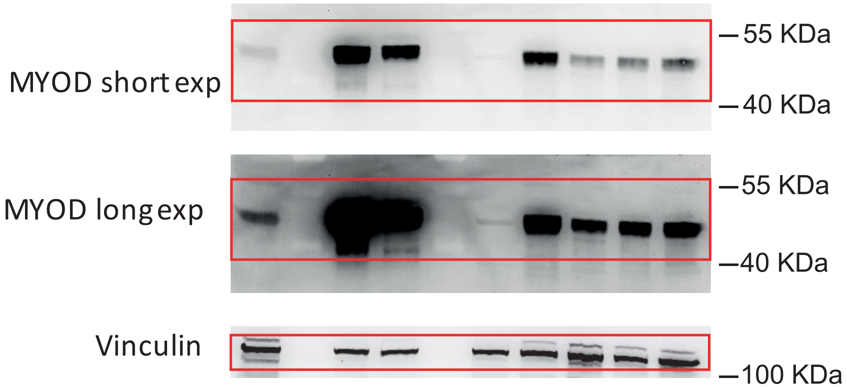

Figure S2b

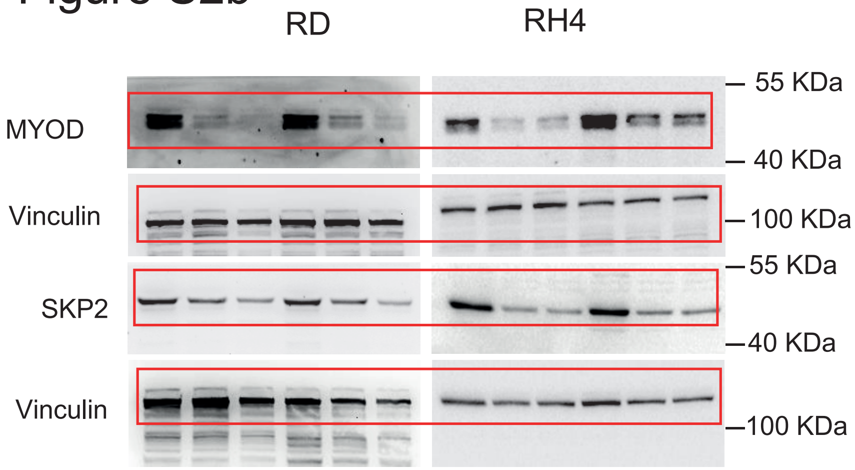

Figure S2d

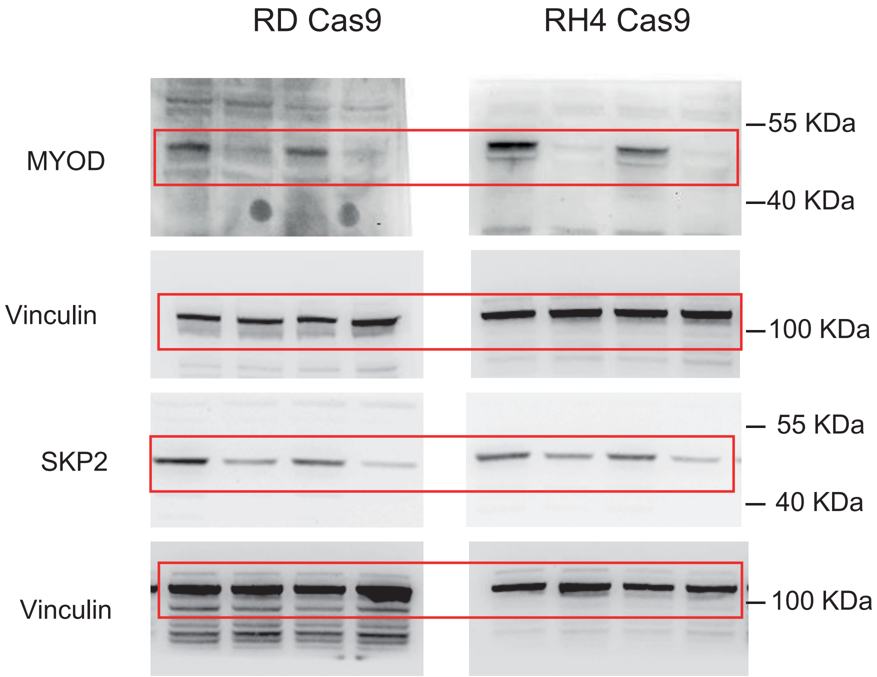

Figure S3b

293T

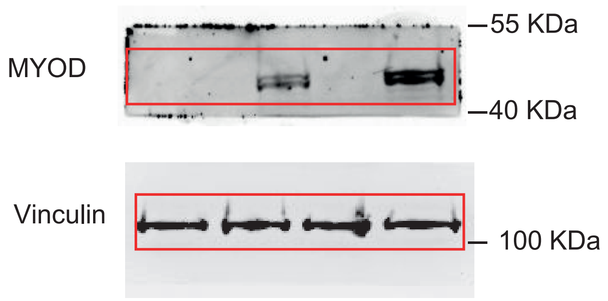

Figure S4c

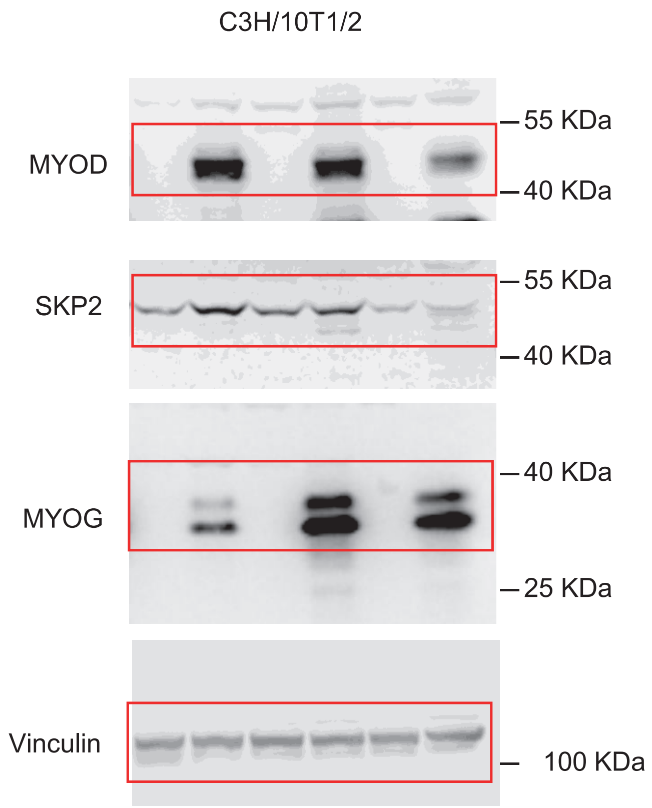
C3H/10T1/2
MYOD
55 KDa
40 KDa
SKP2
55 KDa
40 KDa
MYOG
40 KDa
25 KDa
Vinculin
100 KDa

Figure S5a

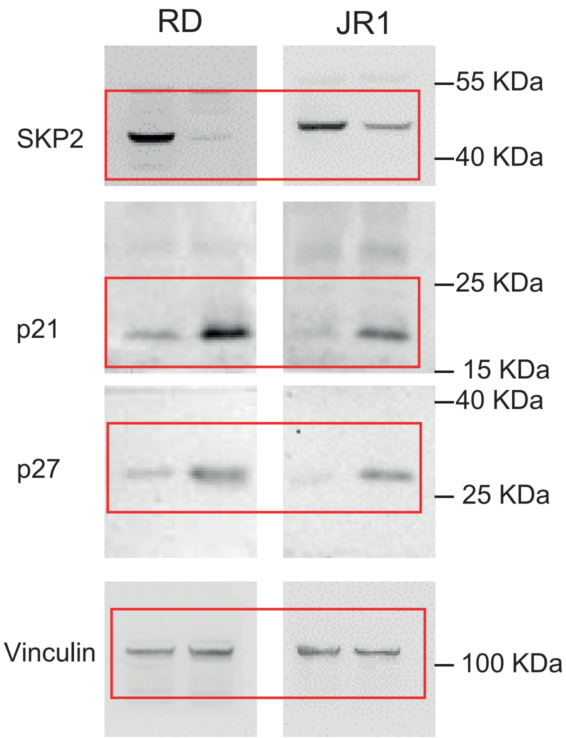

Figure S5b

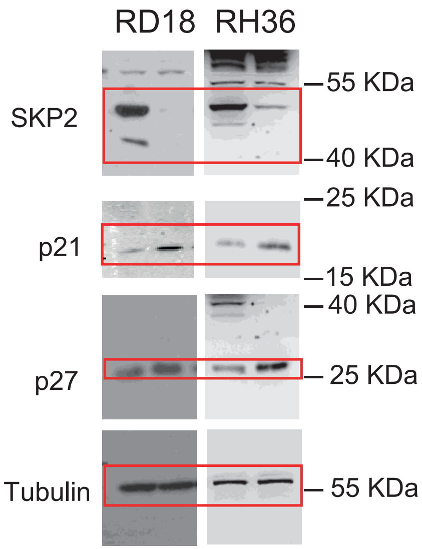

Figure S5d

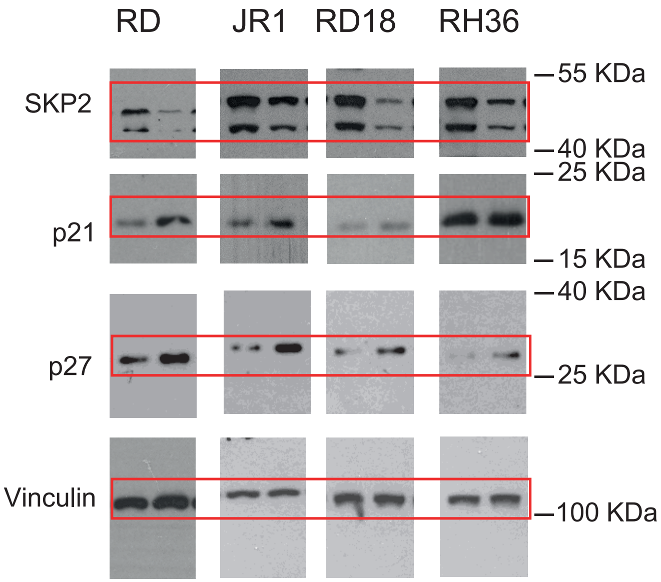

Figure S5f

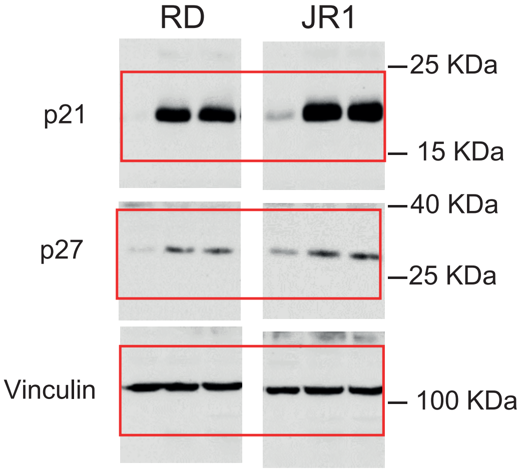

Figure S5g

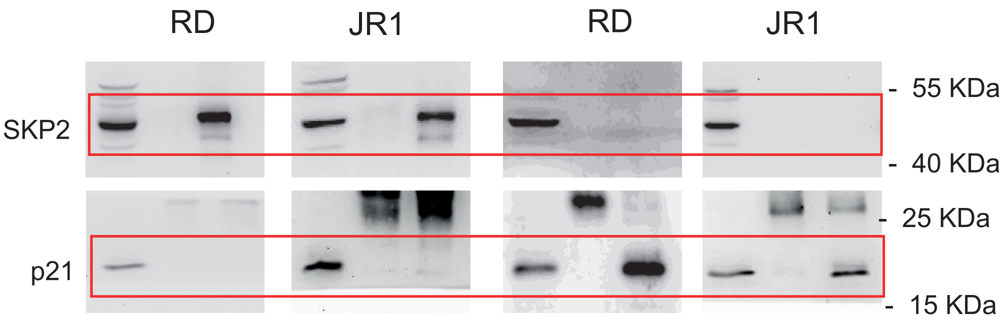

Figure S6a

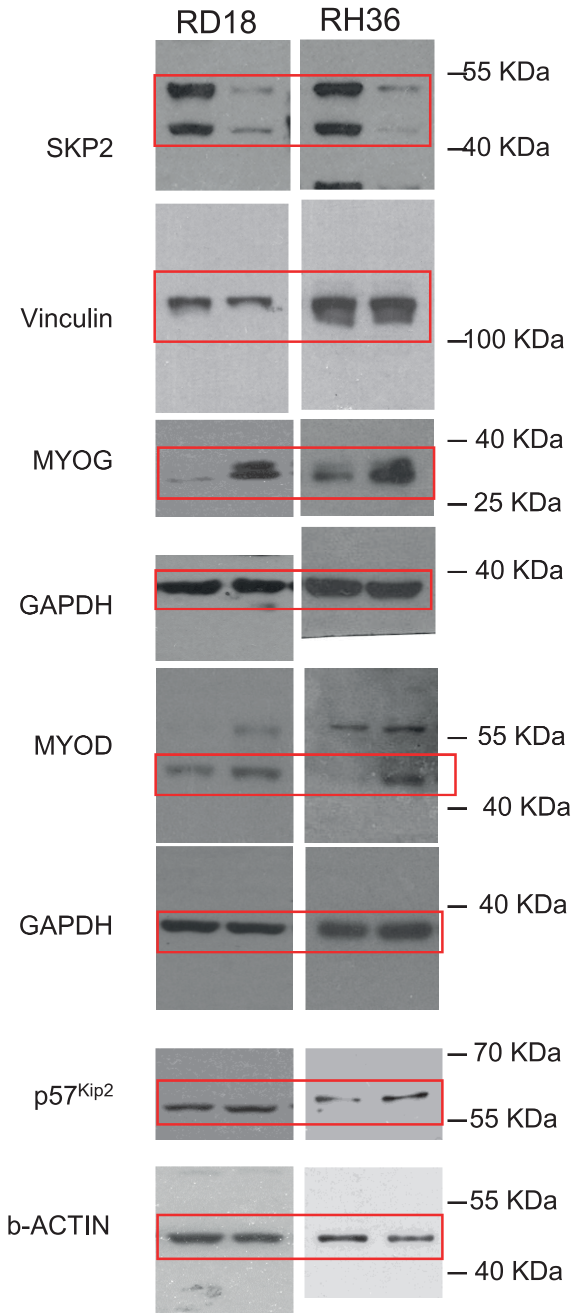
RD18
RH36
SKP2
Vinculin
MYOG
GAPDH
MYOD
GAPDH
p57Kip2
b-ACTIN
55 KDa
40 KDa
100 KDa
40 KDa
25 KDa
40 KDa
55 KDa
40 KDa
40 KDa
70 KDa
55 KDa
55 KDa
40 KDa

Figure S7a

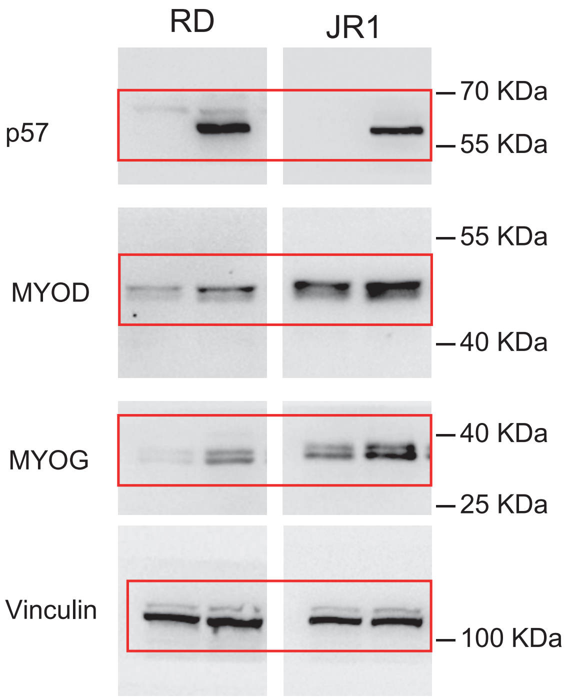
RD
JR1
p57
MYOD
MYOG
Vinculin
70 KDa
55 KDa
55 KDa
40 KDa
40 KDa
25 KDa
100 KDa

Figure S8a

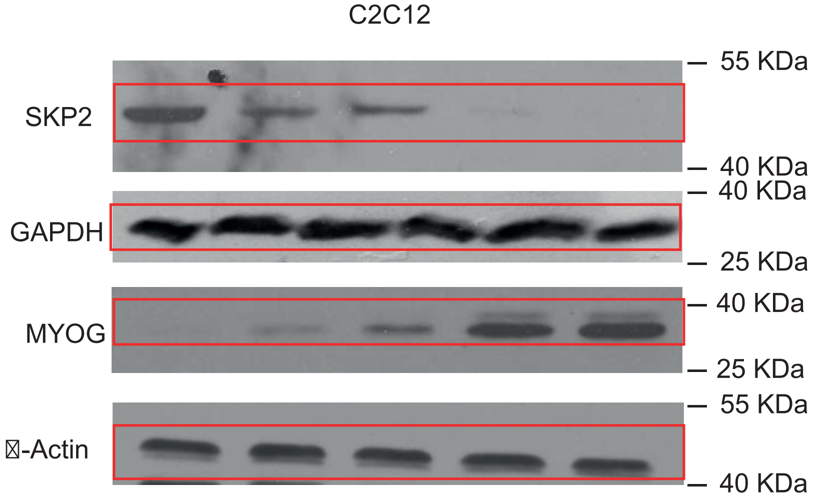

Figure S8b

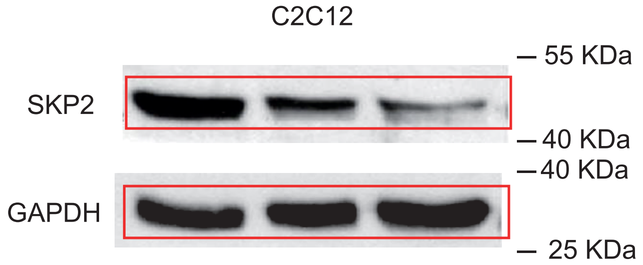

Figure S8d

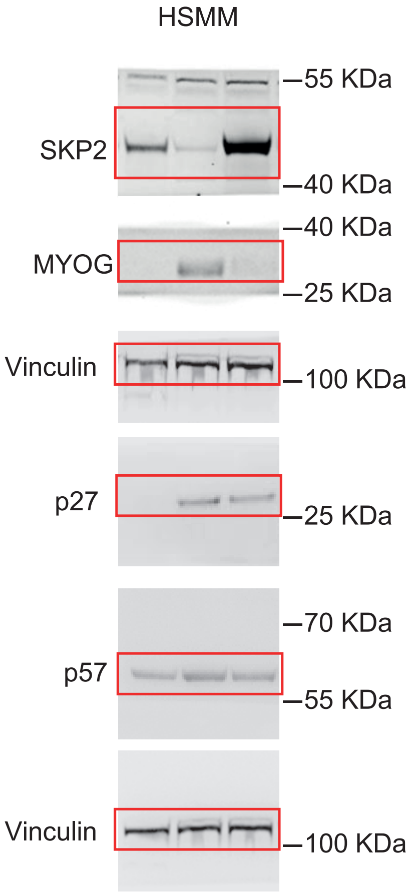

Figure S10e

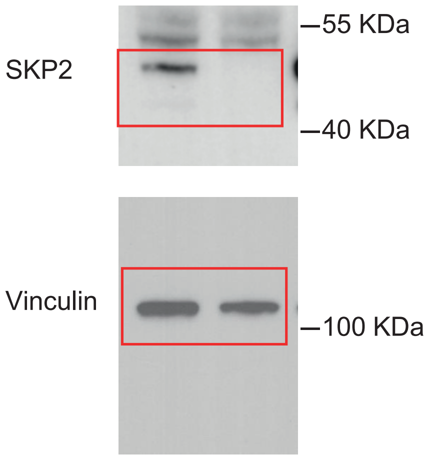

Figure S11h

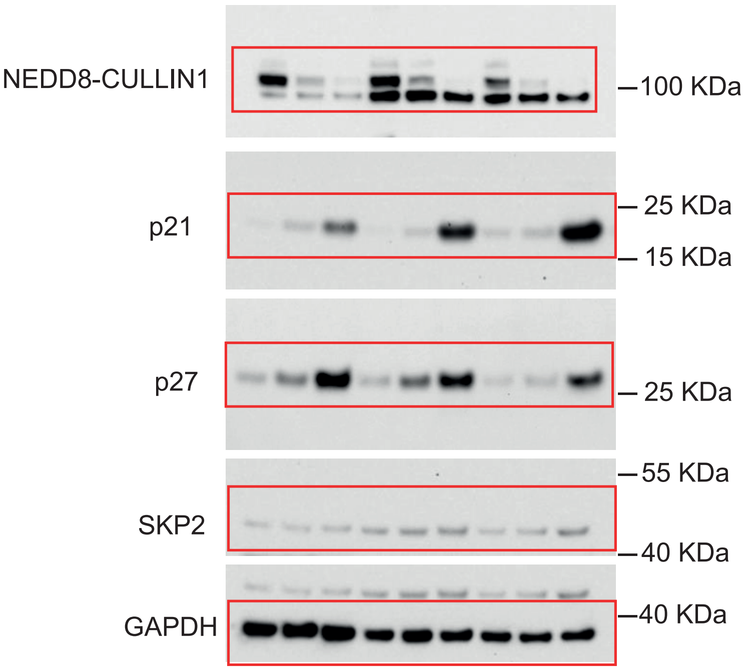

NEDD8-CULLIN1
100 KDa
p21
25 KDa
15 KDa
p27
25 KDa
SKP2
55 KDa
40 KDa
GAPDH
40 KDa
