# Supplementary material for: MYOD-SKP2 axis boosts tumorigenesis in fusion negative rhabdomyosarcoma by preventing differentiation through p57Kip2 targeting
Source: Nat Commun. 2023 Dec 15;14:8373. doi: 10.1038/s41467-023-44130-0 (PMC10724275; doi:10.1038/s41467-023-44130-0)
Supplement: Supplementary file 2 — Description of Additional Supplementary Files [file 41467_2023_44130_MOESM2_ESM.pdf]

Description of Additional Supplementary Files

Supplementary Data 1. Genes up-regulated (log2Fold Change > 1.3) and down-regulated (log2Fold Change<0.7) in RD and JR1 cells.

Supplementary Data 2. RD and JR1 commonly modulated genes in HALLMARK_MYOGENESIS, FRIDMAN_SENESCENCE_DN and RAMALHO_STEMNESS_UP gene sets.
