# Supplementary material for: MYOD-SKP2 axis boosts tumorigenesis in fusion negative rhabdomyosarcoma by preventing differentiation through p57Kip2 targeting
Source: Nat Commun. 2023 Dec 15;14:8373. doi: 10.1038/s41467-023-44130-0 (PMC10724275; doi:10.1038/s41467-023-44130-0)
Supplement: Supplementary file 4 — Reporting Summary [file 41467_2023_44130_MOESM4_ESM.pdf]

nature portfolio

Corresponding author(s): Rota R

Last updated by author(s): Oct 30, 2023

Reporting Summary

Nature Portfolio wishes to improve the reproducibility of the work that we publish. This form provides structure for consistency and transparency in reporting. For further information on Nature Portfolio policies, see our Editorial Policies and the Editorial Policy Checklist.

Statistics

For all statistical analyses, confirm that the following items are present in the figure legend, table legend, main text, or Methods section.

n/a	Confirmed	
☐	☒	The exact sample size (*n*) for each experimental group/condition, given as a discrete number and unit of measurement
☐	☒	A statement on whether measurements were taken from distinct samples or whether the same sample was measured repeatedly
☐	☒	The statistical test(s) used AND whether they are one- or two-sided<br>*Only common tests should be described solely by name; describe more complex techniques in the Methods section.*
☐	☒	A description of all covariates tested
☐	☒	A description of any assumptions or corrections, such as tests of normality and adjustment for multiple comparisons
☐	☒	A full description of the statistical parameters including central tendency (e.g. means) or other basic estimates (e.g. regression coefficient) AND variation (e.g. standard deviation) or associated estimates of uncertainty (e.g. confidence intervals)
☐	☒	For null hypothesis testing, the test statistic (e.g. *F*, *t*, *r*) with confidence intervals, effect sizes, degrees of freedom and *P* value noted<br>*Give P values as exact values whenever suitable.*
☒	☐	For Bayesian analysis, information on the choice of priors and Markov chain Monte Carlo settings
☒	☐	For hierarchical and complex designs, identification of the appropriate level for tests and full reporting of outcomes
☐	☒	Estimates of effect sizes (e.g. Cohen's *d*, Pearson's *r*), indicating how they were calculated

Our web collection on statistics for biologists contains articles on many of the points above.

Software and code

Policy information about availability of computer code

Data collection	Image acquisition for the determination of G150 by Celigo Imaging Cytometer (Nexcelom Bioscience, Lawrence, MA, USA); the measurement of Caspase-3/7 activity and luciferase activity assay by EnSpire Multimode Plate Reader (PerkinElmer, Waltham, MA, USA); the mRNA expression levels by Applied Biosystems 7900HT Fast RealTime PCRSystem (Applied Biosystems, Waltham, MA, USA); light microscopy imaging was performed by Olympus Digital Camera XC50 (Olympus Corporation, Shinjuku-ku, Tokyo, Japan); flow cytometry experiments were performed on FACSCantoll equipped with a FACSDiva 6.1CellQuestTM software (Becton Dickinson Instrument, San Jose, CA, USA); B-galactosidase staining assay images and fluorescence images were acquired with Leica DMi8 microscope (Leica microsystems, Mannhein, Germany); Proximity Ligation Assay images were acquired by Olympus FV3000 confocal microscopy with Olympus FV315S-SW image 26 acquisition software; the light microscopy immunohistochemistry imaging was performed on a Nikon 28 E600 light microscope equipped with NIS Elements BR software, using 20x objective; image acquisition for western blotting was performed on iBright FL1500 Imaging System equipped with iBright Analysis Software desktop version.
Data analysis	Poly-A selected RNA libraries were prepared using the NEBNext Ultra II Directional RNA Library Prep kit and sequenced on an Illumina NovaSeq6000 System (2x150bp). Reads were aligned to the GRCh38 reference genome using STAR version 2.7.9a, and gene expression was calculated as Transcript Per Million (TPM) reads using RSEM version 1.3.2 via a maximum likelihood estimation framework. Gene Set Enrichment Analysis (GSEA) was performed using rank lists of log2-fold change in TPM, comparing each shSKP2 condition to its paired shSCR control RNA-seq experiment. z score normalization has been performed to visualize differentially expressed genes as heatmap. ChIP-seq libraries were prepared using the NEBNext Ultra II DNA Library Prep kit and sequenced on an Illumina NovaSeq6000 (2x150bp). Reads were aligned to the GRCh38 reference genome using BWA version 0.7.17 and samples were normalized to reads per million mapped Drosophila spike-in reads, previously aligned on dm3 reference genome. Peak calling was performed using MACS3 and mapping artifacts were removed by excluding regions listed in ENCODE exclusion list (ENCFF356LFX). Heatmaps and average density profiles were generated using deepTools by plotting H3K27ac and MYOD ChIP-seq read coverages over MYOD-bound genomic regions. ChIP-seq enrichment peaks of public dataset

were visualized with IGV (2.16.2). Proximity-ligation and library preparation for HiChIP were sequenced on an Illumina NovaSeq System (2x150bp). To obtain valid read pairs, paired-end reads were mapped to the human (hg38) or mouse (mm10) reference genome using Bowtie2 within the HiC-Pro pipeline. Reads were then filtered for read pairs with contact range >1000bp and final read pairs were visualized on Juicebox. All downstream analyses requiring sub-matrix extraction from .hic files were performed using Juicer and strawr. Contact maps and Aggregated Peak Analysis (APA) plots were used to visualize 3D contacts. Software used for statistical tests include Graphpad Prism (8.4.3) and Microsoft Excel (16.0.10392). Other used softwares were ImageJ (2.0.0), Adobe Photoshop (2022), Adobe Illustrator (2022), BioRender.com; ggplot2 (3.4.4); clusterProfiler.

For manuscripts utilizing custom algorithms or software that are central to the research but not yet described in published literature, software must be made available to editors and reviewers. We strongly encourage code deposition in a community repository (e.g. GitHub). See the Nature Portfolio guidelines for submitting code & software for further information.

Data

Policy information about availability of data

All manuscripts must include a data availability statement. This statement should provide the following information, where applicable:

- Accession codes, unique identifiers, or web links for publicly available datasets
- A description of any restrictions on data availability
- For clinical datasets or third party data, please ensure that the statement adheres to our policy

All the data generated during the current study are available at GEO (Gene Expression Omnibus) with the following accession number GSE241283 (GRCh38 reference genome).
Previously published GEO datasets used in the study were: Affymetrix profiling data (GSE66533, GSE14333, GSE108474, GSE111678, GSE14827, G5E16011, GSE26673, GSE31684, GSE32676, GSE32701, GSE34620, GSE39671, GSE42743, GSE43580, GSE64019, GSE64415, GSE7553, GSE7696, GSE87371, GSE9843, GSE2658, GSE16476, GSE9891, GSE9103, GSE2109, GSE7307 and (Northcott PA. et al., Nature 2017).
ChIP-seq data (GSE83728, GSE137168, GSE29611).
RNA-seq data (GSE52529).
The remaining data are available within the article and Source Data are provided with this paper.

Research involving human participants, their data, or biological material

Policy information about studies with human participants or human data. See also policy information about sex, gender (identity/presentation), and sexual orientation and race, ethnicity and racism.

Reporting on sex and gender	No sex or gender information associated with the tissue samples were requested because the study design did not involve gender-related factors and was only aimed at evaluating the expression of the SKP2 protein in primary tissues sections. Thus, these informations are not relevant in this study.
Reporting on race, ethnicity, or other socially relevant groupings	Characteristics on race, ethnicity, or other socially relevant groupings were not collected because not relevant in this study.
Population characteristics	Formalin-fixed paraffin embedded (FFPE) tissue blocks from cases of fusion negative or fusion positive rhabdomyosarcoma were obtained at the diagnosis in patients aged from 1 to 21 years from department archives of the Pathology Unit of Bambino Gesù Children's Hospital per Institutional Review Board (IRB) approval (see Methods). Biopsy of adjacent normal muscle tissues were done when ethically possible. All tissue sections were de-identified prior to their use in immunohistochemical stains for the manuscript. No clinical information associated with the tissue samples was requested because the study was only aimed at evaluating the expression of the SKP2 protein in primary tissues sections and, thus, no clinical correlations were needed or done.
Recruitment	All FFPE tissue samples were obtained retrospectively. No patients were recruited for this study.
Ethics oversight	Sections of FFPE human samples were obtained from the Pathology Unit of Bambino Gesù Children's Hospital (Rome, Italy) and the approval of the study was obtained in accordance with the Institutional Review Board (IRB) of Bambino Gesù Children's Hospital, IRCCS, Rome, Italy (OPBG; Authorization 120 LB, 02/10/2015). Written informed consent was obtained from all patients. No compensation has been paid.

Note that full information on the approval of the study protocol must also be provided in the manuscript.

Field-specific reporting

Please select the one below that is the best fit for your research. If you are not sure, read the appropriate sections before making your selection.

☒ Life sciences ☐ Behavioural & social sciences ☐ Ecological, evolutionary & environmental sciences

For a reference copy of the document with all sections, see nature.com/documents/nr-reporting-summary-flat.pdf

Life sciences study design

All studies must disclose on these points even when the disclosure is negative.

Sample size	We estimated the sample size considering no significant variation within each group of data. The principle of using the smallest sample size

	possible was adopted in planning the animal experiments. We estimated the sample size in order to detect a difference in averages of 2 standard deviations at the 0.05 level of significance with an 80% power. No sample-size calculation was performed for the in vitro experiments. Each condition was analyzed with three biological replicates, a standard for the experiments performed to account for reasonable range of variability among samples.
Data exclusions	No data was excluded from the study.
Replication	Experiments were repeated at least in three biological replicates unless stated otherwise and all attempts at replication were successful.
Randomization	Experimental mice were randomized into the experimental groups following tumor inoculation to ensure equal mean tumor volume per condition before pharmacological treatments. For the experiments using shRNA, no randomization was applied after cell inoculation since mice were grouped based on the injected cell type to have shSCR vs shSKP2. For studies not involving animals, no randomization was required.
Blinding	In in vivo pharmacological studies, blinding was performed only at the start of the experiments, when mice were randomized into the experimental groups following tumor inoculation before drug treatment. However, the medium size of the tumors of each group was similar. Tumor size data were collected in a blinding manner. For all the other subsequent steps, blinding was not feasible given the study design and the labeling requirements for the operative procedures of the facilities. IHC stainings were performed in blinding manner.

Reporting for specific materials, systems and methods

We require information from authors about some types of materials, experimental systems and methods used in many studies. Here, indicate whether each material, system or method listed is relevant to your study. If you are not sure if a list item applies to your research, read the appropriate section before selecting a response.

Materials & experimental systems

n/a	Involved in the study	
☐	☒	Antibodies
☐	☒	Eukaryotic cell lines
☒	☐	Palaeontology and archaeology
☐	☒	Animals and other organisms
☒	☐	Clinical data
☒	☐	Dual use research of concern
☒	☐	Plants

Methods

n/a	Involved in the study	
☐	☒	ChIP-seq
☐	☒	Flow cytometry
☒	☐	MRI-based neuroimaging

Antibodies

Antibodies used

SKP2 (H-435) Santa Cruz Biotechnology Cat# sc-7164, RRID:AB_ 2187650, Lot: F1714, 1:250 (WB)
MyoD (5-8A) Santa Cruz Biotechnology Cat# sc-32758, RRID:AB_2148870, Lot: H2622, 1:200 (WB)
pMYOD (Ser-200) Santa Cruz Biotechnology Cat# sc-101741, RRID:AB_ 2148764, Lot: E0708 1:200 (WB)
p27 Kip1 (F-8) Santa Cruz Biotechnology Cat# sc-1641, RRID:AB_ 628074, Lot: H0416, 1:500 (WB)
Myosin Heavy Chain DSHB Cat# MF 20, RRID:AB_2147781, Lot: 02/13/2020, 1:100 (IF); 1:50 (IHC)
p21 Waf1/Cipl (12D1) Cell Signaling Technology Cat# 2947, RRID:AB_823586, Lot: 11; 1:1000 (WB); 1:50 (IHC), 1:1000 (weight:weight) (IP)
Nedd8 19E3 Cell Signaling Technology Cat# 2754, RRID:AB_659972, Lot: 5, 1:1000 (WB)
MYOG DSHB Cat# F5D, RRID:AB_2146602, Lot: 12/17/2018, 1:200 (WB); 1:100 (IHC)
p57 Kip2 BD Biosciences Cat# 556346, RRID:AB_396375, Lot: 1134625, 1:500 (WB)
GAPDH (D16H11) Cell Signaling Technology Cat# 5174, RRID:AB_10622025, Lot: 8, 1:2000 (WB)
b-ACTIN (C4) Santa Cruz Biotechnology Cat# sc-47778, RRID:AB_626632, Lot: I2822, 1:1000 (WB)
a-Tubulin (DM1A) Novus Biologicals Cat# NB100_680, RRID:AB_ 1218281, Lot: G-2, 1:5000 (WB)
Vinculin (hVIN-1) Sigma Cat# V9131, RRID:AB_477629, Lot: 079M475, 1:5000 (WB)
HRP (Horseradish peroxidase) anti-rabbit Cell Signaling Technology Cat# 7074P2, RRID:AB_2099233, Lot:30, 1:5000 (WB)
HRP (Horseradish peroxidase) anti-mouse Cell Signaling Technology Cat# 7076, RRID:AB_330924, Lot: 32, 1:5000 (WB)
SKP2 (8D9) Thermofisher Scientific Cat# 32-3300, RRID:AB_2533074, Lot: RC2177202A, 1:1000 (weight:weight) (IP), 1:50 (IHC)
p27 Kip1 (SX53G8.5) Santa Cruz Biotechnology Cat# sc-53871, RRID:AB_ 785029, Lot: J2511, 1:50 (IHC)
p57 Kip2 (KP39) Santa Cruz Biotechnology Cat# sc-56341, RRID:AB_ 785045, Lot: K2119, 1:100 (IHC)
Cleaved Caspase 3 Cell Signaling Technology Cat# 9661, RRID:AB_2341188, Lot: 47, 1:300 (IHC)
Ki67 (MIB-1) Dako Cat# IR626, RRID:AB_2890068, Lot: 41340767, ready to use (IHC)
H3K27Ac Active motif Cat# 39133, RRID:AB_2561016, Lot: 31521015, 1:1 (weight:weight) (ChIP)
MYOD (D8G3) Cell Signaling Technology Cat# 13812, RRID:AB_2798320, Lot: 1, 1:10 (weight:weight) (ChIP)
Drosophila Spike-in Active motif Cat# 61686, RRID:AB_2737370, Lot: 23521010, 1:2 (weight:weight) (ChIP)
Goat anti-Mouse IgG Alexa Fluor™ 488, Invitrogen Cat#A11017, RRID:AB_143160, Lot:2454774, 1:1000 (IF)
Goat anti-Mouse IgG Alexa Fluor™ 555, Invitrogen Cat#A21425, RRID:AB_2535846, Lot: 2184319, 1:1000 (IF)

Validation

All Antibodies listed above are commercially available and validated by correspondent suppliers, which is described in the manufacturer's website:

- Rabbit anti-mouse and human SKP2 (H-435) antibody is validated for WB: https://www.scbt.com/it/p/skp2-p45-antibody-h-435#citations
- Mouse anti-mouse and human MyoD (5.8A) antibody is validated for WB: https://datasheets.scbt.com/sc-32758.pdf
- Rabbit anti-human pMYOD (Ser-200) antibody is validated for WB: https://datasheets.scbt.com/sc-101741.pdf
- Mouse anti-human p27 Kip1 (F-8) antibody is validated for WB: https://www.scbt.com/it/p/p27-antibody-f-8
- Mouse anti-human Myosin Heavy Chain MF 20 antibody is validated for IF and IHC: https://dshb.biology.uiowa.edu/MF-20
- Rabbit anti-human p21 Waf1/Cip1 (12D1) antibody is validated for WB, IHC and IP: https://www.cellsignal.com/products/primary-antibodies/p21-waf1-cip1-12d1-rabbit-mab/2947
- Rabbit anti-human Nedd8 19E3 antibody is validated for WB: https://www.cellsignal.com/products/primary-antibodies/nedd8-19e3-rabbit-mab/2754
- Mouse anti-human and mouse MYOG (F5D) antibody is validated for IHC and WB: https://dshb.biology.uiowa.edu/F5D
- Mouse anti-human p57 Kip2 antibody is validated for WB: https://www.bdbiosciences.com/en-us/products/reagents/western-blotting-and-molecular-reagents/western-blot-reagents/purified-mouse-anti-human-p57-kip2.556346
- Rabbit anti-human and mouse GAPDH (D16H11) antibody is validated for WB: https://www.cellsignal.com/products/primary-antibodies/gapdh-d16h11-xp-rabbit-mab/5174
- Mouse anti-human and mouse b-ACTIN (C4) antibody is validated for WB: https://www.scbt.com/it/p/beta-actin-antibody-c4
- Mouse anti-human aTubulin (DM1A) antibody is validated for WB: https://www.novusbio.com/products/alpha-tubulin-antibody-dm1a_nb100-690
- Mouse anti- human and mouse Vinculin (hVIN-1) antibody is validated for WB: https://www.sigmaaldrich.com/IT/it/product/sigma/v9131
- Goat ant-rabbit HRP (Horseradish peroxidase) antibody is validated for WB: https://www.cellsignal.com/products/secondary-antibodies/anti-rabbit-igg-hrp-linked-antibody/7074
- Horse anti-mouse HRP antibody is validated for WB: https://www.cellsignal.com/products/secondary-antibodies/anti-mouse-igg-hrp-linked-antibody/7076
- Mouse anti-human SKP2 (8D9) antibody is validated for IP: https://www.thermofisher.com/antibody/product/SKP2-Antibody-clone-SKP2-8D9-Monoclonal/32-3300
- Mouse anti-human p27 Kip1 (SX53G8.5) antibody is validated for IHC: https://www.scbt.com/p/p27-antibody-sx53g8-5
- Mouse anti-human p57 Kip2 (KP39) antibody is validated for IHC: https://www.scbt.com/it/p/p57-antibody-kp39
- Rabbit anti-human Cleaved Caspase-3 (Asp175) antibody is validated for IHC: https://www.cellsignal.com/products/primary-antibodies/cleaved-caspase-3-asp175-antibody/9661
- Mouse anti-human Ki67 antibody is validated for IHC: https://www.agilent.com/en/product/immunohistochemistry/antibodies-controls/primary-antibodies/ki-67-antigen-(autostainer-link-48)-76356
- Rabbit anti-human H3K27Ac antibody is validated for ChIP, ChIP-seq: https://www.activemotif.com/catalog/details/39133/histone-h3-acetyl-lys27-antibody-pab
- Rabbit anti-human MYOD (D8G3) antibody is validated for ChIP: https://www.cellsignal.com/products/primary-antibodies/myod1-d8g3-xp-rabbit-mab/13812
- Rabbit anti-Drosophila Spike-in antibody is validated for ChIP, ChIP-seq: https://www.activemotif.com/catalog/1091/chip-normalization
- Goat anti-Mouse IgG Alexa Fluor™ 488 secondary antibody is validated for immunofluorescence: https://www.thermofisher.com/antibody/product/Goat-anti-Mouse-IgG-H-L-Cross-Adsorbed-Secondary-Antibody-Polyclonal/A-11017
-Goat anti-Mouse IgG Alexa Fluor™ 555 secondary antibody is validated for immunofluorescence: https://www.thermofisher.com/antibody/product/Goat-anti-Mouse-IgG-H-L-Cross-Adsorbed-Secondary-Antibody-Polyclonal/A-21425

Eukaryotic cell lines

Policy information about cell lines and Sex and Gender in Research

Cell line source(s)

RD and RH30 were obtained from ATCC (Rockville, MD, USA), RD18 cells were a gift of C. Ponzetto (Department of Oncology, University of Turin, Turin, Italy). RH2, JR1, RH36 and RH4 cells were provided by P. Houghton. Human Skeletal Muscle Myoblasts (HSMM) and Normal Human Lung Fibroblast (NHLF) were purchased from Lonza (Walkersville, MD, USA). C2C12 and C3H/10T1/2 were purchased from ATCC(Rockville, MD, USA). Orthotopic patient-derived xenograft (o-PDX) cells SJRHB011_YC, SJRHB012_YC and SJRHB012_ZC were obtained through the Childhood Solid Tumor Network (CSTN) at St. Jude Children's Hospital (Memphis, TN, USA).

Authentication

The cell lines used were authenticated by STR profiling.

Mycoplasma contamination

Cell lines are tested for mycoplasma contamination by PCR every month. All lines to date tested negative.

Commonly misidentified lines
(See ICLAC register)

There were no commonly misidentified cell lines used in this study.

Animals and other research organisms

Policy information about studies involving animals; ARRIVE guidelines recommended for reporting animal research, and Sex and Gender in Research

Laboratory animals

Species: Mus musculus; strain: RRID:IMSR_JAX:005557, NOD.Cg-Prkdcscid Il2rgtm1Wjl/SzJ (NOD SCID Gamma (NSG) mice provided by Charles River, www.criver.com); sex: female; Age: 6-8 weeks. Animals were maintained in sterile conditions, 12h light/12h dark cycle, ambient temperature 18-23° C with 40-60% humidity.

Wild animals | Study did not involve wild animals.

Reporting on sex | Species: Mus musculus; strain: RRID:IMSR_JAX:005557, NOD.Cg-Prkdcscid Il2rgtm1Wjl/SzJ (NOD SCID Gamma (NSG) mice provided by Charles River, www.criver.com); sex: female. The xenograft studies involved female mice as in our previous published work (Pomella S et al., Nature Communication 2021). Female mice allow the housing of maximum five mice per cage.

Field-collected samples | Study did not involve field collected samples.

Ethics oversight | All animal experiments were performed in accordance with the Guidelines for Animal Care and Use of the National Institutes of Health and approved by the Institutional Animal Care and Use Committee (IACUC) at the University of Texas Health Science Center, San Antonio, Texas (protocol number 20150015AR) and in accordance with the European Communities Council Directive N. 2010/63/EU, the Italian Ministry of Health guidelines (DL 26/2014) and approved by the Italian Ministry of Health for the Children's Hospital Bambino Gesu/Plaisant animal facility of Castel Romano in Rome, Italy (protocol number 88/2016-PR).

Note that full information on the approval of the study protocol must also be provided in the manuscript.

Plants

Seed stocks | *Report on the source of all seed stocks or other plant material used. If applicable, state the seed stock centre and catalogue number. If plant specimens were collected from the field, describe the collection location, date and sampling procedures.*

Novel plant genotypes | *Describe the methods by which all novel plant genotypes were produced. This includes those generated by transgenic approaches, gene editing, chemical/radiation-based mutagenesis and hybridization. For transgenic lines, describe the transformation method, the number of independent lines analyzed and the generation upon which experiments were performed. For gene-edited lines, describe the editor used, the endogenous sequence targeted for editing, the targeting guide RNA sequence (if applicable) and how the editor was applied.*

Authentication | *Describe any authentication procedures for each seed stock used or novel genotype generated. Describe any experiments used to assess the effect of a mutation and, where applicable, how potential secondary effects (e.g. second site T-DNA insertions, mosiacism, off-target gene editing) were examined.*

ChIP-seq

Data deposition

- [x] Confirm that both raw and final processed data have been deposited in a public database such as GEO.
- [x] Confirm that you have deposited or provided access to graph files (e.g. BED files) for the called peaks.

Data access links
May remain private before publication. | https://www.ncbi.nlm.nih.gov/geo/query/acc.cgi?acc=GSE241283

Files in database submission |
GSM7720561RD_shSCR, H3K27ac, ChIP
GSM7720562 RD_shSKP2, H3K27ac, ChIP
GSM7720563 RD_shSCR, MYOD, ChIP
GSM7720564 RD_shSKP2, MYOD, ChIP
GSM7843893 RD, H3K27ac, HiChIP
GSM7843894 RH4, H3K27ac, HiChIP

Genome browser session
(e.g. UCSC) | No longer applicable.

Methodology

Replicates | One replicate was used for each condition.

Sequencing depth | ChIP-seq libraries were prepared using the NEBNext Ultra II DNA Library Prep kit and sequenced on an Illumina NovaSeq6000 (2x150bp). Reads were aligned to the GRCh38 reference genome using BWA version 0.7.17 and samples were normalized to reads per million mapped Drosophila spike-in reads, previously aligned on dm3 reference genome. Instrument mode: Illumina NextSeq 500 Read length: 75 Single end. Proximity-ligation and library preparation for Hi-ChIP were sequenced on an Illumina NovaSeq System (2x150bp). Paired-end reads were mapped to the human (hg38) or mouse (mm10) reference genome using Bowtie2 within the HiC-Pro pipeline. Reads were then filtered for read pairs with contact range >1000bp and final read pairs were visualized on Juicebox.

Antibodies |
H3K27Ac Active motif Cat# 39133, RRID:AB_2561016
MYOD (D8G3) Cell Signaling Technology Cat# 13812, RRID:AB_2798320
Drosophila Spike-in Active motif Cat# 61686, RRID:AB_2737370

Peak calling parameters | Peak calling was performed using MACS3 (version v3.0.0, https://github.com/taoliu/MACS) using "narrow" mode for all targets reported in this paper, as they form sharp genomic peaks. Parameters for MACS3 usage: [--format BAM--controlinput.bam--keep-dup all--pvalue 0.00001] . Regions called as peaks which are known to be spurious mapping artifacts were removed before any further analysis (reference location or sites black-listed by ENCODE exclusion list (ENCFF356LFX), https://www.encodeproject.org/files/ENCFF356LFX/).

Data quality	Described in peak calling parameters.
Software	Heatmaps and average density profiles were generated using deepTools by plotting H3K27ac and MYOD ChIP-seq read coverages over MYOD-bound genomic regions. ChIP-seq enrichment peaks of public datasets were visualized with IGV. Hi-ChIP paired-end reads were mapped to the human (hg38) or mouse (mm10) reference genome using Bowtie2 within the HiC-Pro pipeline. Final read pairs were visualized on Juicebox. All downstream analyses requiring sub-matrix extraction from .hic files were performed using Juicer and strawr. Contact maps and Aggregated Peak Analysis (APA) plots were used to visualize 3D contacts.

Flow Cytometry

Plots

Confirm that:

- [x] The axis labels state the marker and fluorochrome used (e.g. CD4-FITC).
- [x] The axis scales are clearly visible. Include numbers along axes only for bottom left plot of group (a 'group' is an analysis of identical markers).
- [ ] All plots are contour plots with outliers or pseudocolor plots.
- [x] A numerical value for number of cells or percentage (with statistics) is provided.

Methodology

Sample preparation	RD, JR1, RD18 and RH36 human fusion negative rhabdomyosarcoma cells were harvested by trypsinization, washed in cold phosphate buffered saline (PBS), fixed in cold 50% PBS and 50% acetone/methanol (1:4 v/v) for at least 1h. Fixed cells were pelleted 5 minutes 1500 rpm and alcoholic fixative removed. Pellet was stained in the dark with a solution of 50 µg/ml propidium iodide (PI) (ThermoFisher Scientific, Rockford, USA) and 50 µg/ml RNase (Sigma-Aldrich, St Louis, MO, USA) for 30 min at room temperature.
Instrument	BD FACS CantoII (Becton Dickinson Instrument, San Jose, CA, USA).
Software	BD FACSDiva 6.1 CellQuestTM software (Becton Dickinson Instrument, San Jose, CA, USA).
Cell population abundance	10.000 events were analyzed for each experimental condition. No sorting experiments were performed.
Gating strategy	The gating strategy for Figure 3 e, f, m and Supplementary Figure 5 i, j:<br>1) in the forward scatter and side scatter (FSC-A/SSC-A) all the acquired events were gated to analyze only live cells.<br>2) in the forward scatter (FSC-A/FSC-W) and side scatter (SSC-A/SSC-W) all the events were gated to analyze only singlets population to avoid possible bias due to doublets.<br>3) based on PI incorporation (DNA amount) to identify cell cycle phases.

- [x] Tick this box to confirm that a figure exemplifying the gating strategy is provided in the Supplementary Information.
